# Single Particle Automated Raman Trapping Analysis of Breast Cancer Cell-Derived Extracellular Vesicles as Cancer Biomarkers

**DOI:** 10.1021/acsnano.1c07075

**Published:** 2021-11-04

**Authors:** Jelle Penders, Anika Nagelkerke, Eoghan M. Cunnane, Simon V. Pedersen, Isaac J. Pence, R. Charles Coombes, Molly M. Stevens

**Affiliations:** † Department of Materials, Imperial College London, London SW7 2AZ, United Kingdom; ‡ Department of Bioengineering, Imperial College London, London SW7 2AZ, United Kingdom; § Institute of Biomedical Engineering, Imperial College London, London SW7 2AZ, United Kingdom; ∥ Department of Surgery and Cancer, Hammersmith Hospital, Imperial College, London W120HS, United Kingdom

**Keywords:** diagnostics, cancer, spectroscopy, spectroscopic, confocal, exosomes, extracellular vesicles

## Abstract

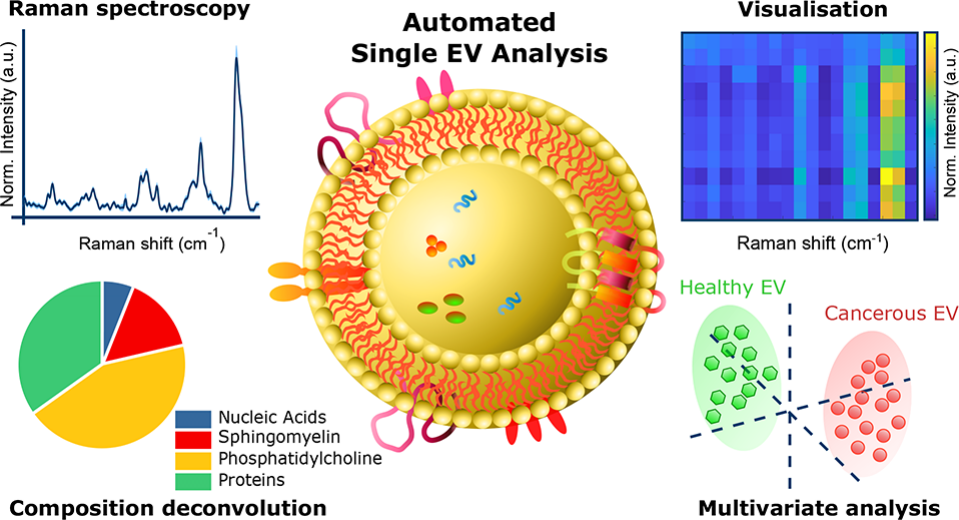

Extracellular vesicles (EVs) secreted by cancer cells provide an important insight into cancer biology and could be leveraged to enhance diagnostics and disease monitoring. This paper details a high-throughput label-free extracellular vesicle analysis approach to study fundamental EV biology, toward diagnosis and monitoring of cancer in a minimally invasive manner and with the elimination of interpreter bias. We present the next generation of our single particle automated Raman trapping analysis—SPARTA—system through the development of a dedicated standalone device optimized for single particle analysis of EVs. Our visualization approach, dubbed dimensional reduction analysis (DRA), presents a convenient and comprehensive method of comparing multiple EV spectra. We demonstrate that the dedicated SPARTA system can differentiate between cancer and noncancer EVs with a high degree of sensitivity and specificity (>95% for both). We further show that the predictive ability of our approach is consistent across multiple EV isolations from the same cell types. Detailed modeling reveals accurate classification between EVs derived from various closely related breast cancer subtypes, further supporting the utility of our SPARTA-based approach for detailed EV profiling.

Breast cancer is one of the most commonly diagnosed types of cancer and the most common type of cancer in women, with over 2 million new cases identified worldwide in 2020.^1^ Breast cancer is characterized by a strong degree of heterogeneity, whereby the receptor status of breast cancer cells has a major impact on prognosis and in determining an effective treatment strategy.^2^ Fast and accurate diagnosis is therefore paramount to effectively treating breast cancer. The most prevalent mode of breast cancer diagnosis is based on physical examination, imaging, and, in the case of suspected malignancy, a biopsy followed by histopathological screening. The primary concerns regarding this approach relate to the invasive nature and the sampling error that can occur with inaccurate biopsy positioning. Furthermore, both X-ray screening and histopathological assessment depend strongly on the individual judgment of trained specialists, with variations between individuals and laboratories frequently reported.^3^ These limitations demonstrate the need for approaches that focus on noninvasive and minimally invasive techniques and reduce interpretation variability and bias.

Raman spectroscopy has several attractive features that would allow its transition into clinical breast cancer diagnostics and disease monitoring.^4−6^ As a light-based technique, Raman spectroscopy is generally label-free and nondestructive, making it possible to directly measure samples *ex* or *in vivo*. It can also be automated, for both data acquisition and analysis, largely eliminating user involvement and bias. Numerous Raman spectroscopy studies have already been conducted on cell lines, primary cells and tissue originating from various types of breast cancer.^5−11^ Such studies have demonstrated that cancerous tissue generally exhibits lipid depletion and protein enrichment,^12,13^ with different lipid to protein ratios depending on the type of breast cancer. Conversely, individual cancer cells have been shown to exhibit an overall increase in lipid content.^3,6^ Raman spectroscopy can therefore potentially reduce user bias, by identifying extracted tissues of cancerous origin. However, extracting tissue via biopsy is an invasive procedure that cannot be performed frequently or routinely for diagnostic purposes or treatment management. Liquid biopsies, most frequently blood samples, have shown increasing promise as a minimally invasive alternative to biopsy.^14^ Blood-borne entities such as circulating tumor cells, circulating tumor DNA, and—of key interest in this study—tumor-derived extracellular vesicles (EVs) are all potential biomarkers.^15,16^

EVs are nanoscale lipid vesicles released by most cell types containing protein and nucleic acid species and can transfer these biomolecules to recipient cells.^17−20^ EVs are one of the tumor cell-secreted factors that aid the formation of the premetastatic niche and directing metastatic organotropism^21−23^ and are increasingly being recognized for their potential as biomarkers of cancer. Several initial studies have been performed that apply Raman spectroscopy to the analysis of EVs from cancerous origin. Spontaneous Raman spectroscopy,^24^ laser trapping Raman spectroscopy,^25−27^ surface-enhanced Raman spectroscopy (SERS),^15,28,37−39,29−36^ and SERS active nanotags^40−44^ have all been applied to acquire fingerprint spectra of cancerous EVs. These previous works show that the spectra of EVs from various cell lines are markedly different and that subpopulations of EVs exist within the cellular secretome that relate to the wide variety of ascribed EV functions. However, revealing the complete complexity of EVs and their fundamental role in cancer biology requires a high-throughput automated technique, to unlock the full benefits of Raman spectroscopy for profiling and ultimately diagnosing cancer.

This study investigates the potential of EVs as biomarkers of breast cancer by applying advances to our recently developed high-throughput single particle automated Raman trapping analysis (SPARTA) platform.^45^ In order to maximize the scientific accuracy and clinical relevance of EV-based cancer biomarkers, a number of key intrinsic and extrinsic experimental parameters must be addressed. The extrinsic factors include the use of appropriate noncancerous cell controls that confirm the ability of the system to differentiate cancerous EVs,^25−31,35^ the characterization of single EVs using a confocal system that limits the spectral signal arising from contaminants,^24,27−30,34,36−38^ the acquisition of high measurement numbers (>100) per sample at high throughput that increases the reliability of the data,^24,25,34−38,26−33^ and the use of an EV isolation method that limits the coprecipitation of cellular proteins and debris.^24,25,36−38,26−31,34,35^ The intrinsic factors relate to the use of a laser trapping Raman spectroscopic system that can analyze label-free EVs in their native state which cannot be achieved using a SERS approach as EVs must be dried on a SERS substrate or functionalized with SERS nanostructures.^15,24,36−38,40−44,28−35^ This study improves upon the state of the art by comprehensively addressing all of these extrinsic and intrinsic study parameters through the use of the SPARTA platform.

We present a core redesign to the SPARTA platform,^45^ from a system integrated into a commercial Raman microspectroscope to a dedicated custom-designed instrument optimized for compositional analysis of EVs. We demonstrate the efficient automated trapping of single EVs derived from noncancerous breast cell types and breast cancer cell lines. A full panel of EVs derived from 11 breast cell types was analyzed, reflecting a variety of origins (noncancerous/primary and metastatic carcinoma) and receptor statuses [human epidermal growth factor receptor 2 (HER2+)/estrogen receptor (ER+)/triple negative]. Over 14 000 individual EV spectra were acquired to capture compositional differences, and for this, a Raman spectral visualization approach dubbed dimensional reduction array (DRA) analysis was utilized. DRA is inspired by previous intensity-based spectra presentations^46,47^ and gene expression arrays for the presentation and interpretation of these complex Raman data sets. A multivariate statistical analysis was performed to establish cancerous/noncancerous classification with high specificity and sensitivity. Crucially, we demonstrate how hyperspectral deconvolution by minimization of the information entropy can be used for demarcation of cancerous EVs at a biomolecular level, without imposing spectral priors, such as reference libraries, on the unmixing. This approach sheds light on the compositional heterogeneity of EVs in exceptional single particle detail. Our dedicated SPARTA system represents an automated and versatile approach to investigating and realizing EVs as cancer biomarkers.

## Results and Discussion

**Figure 1:**
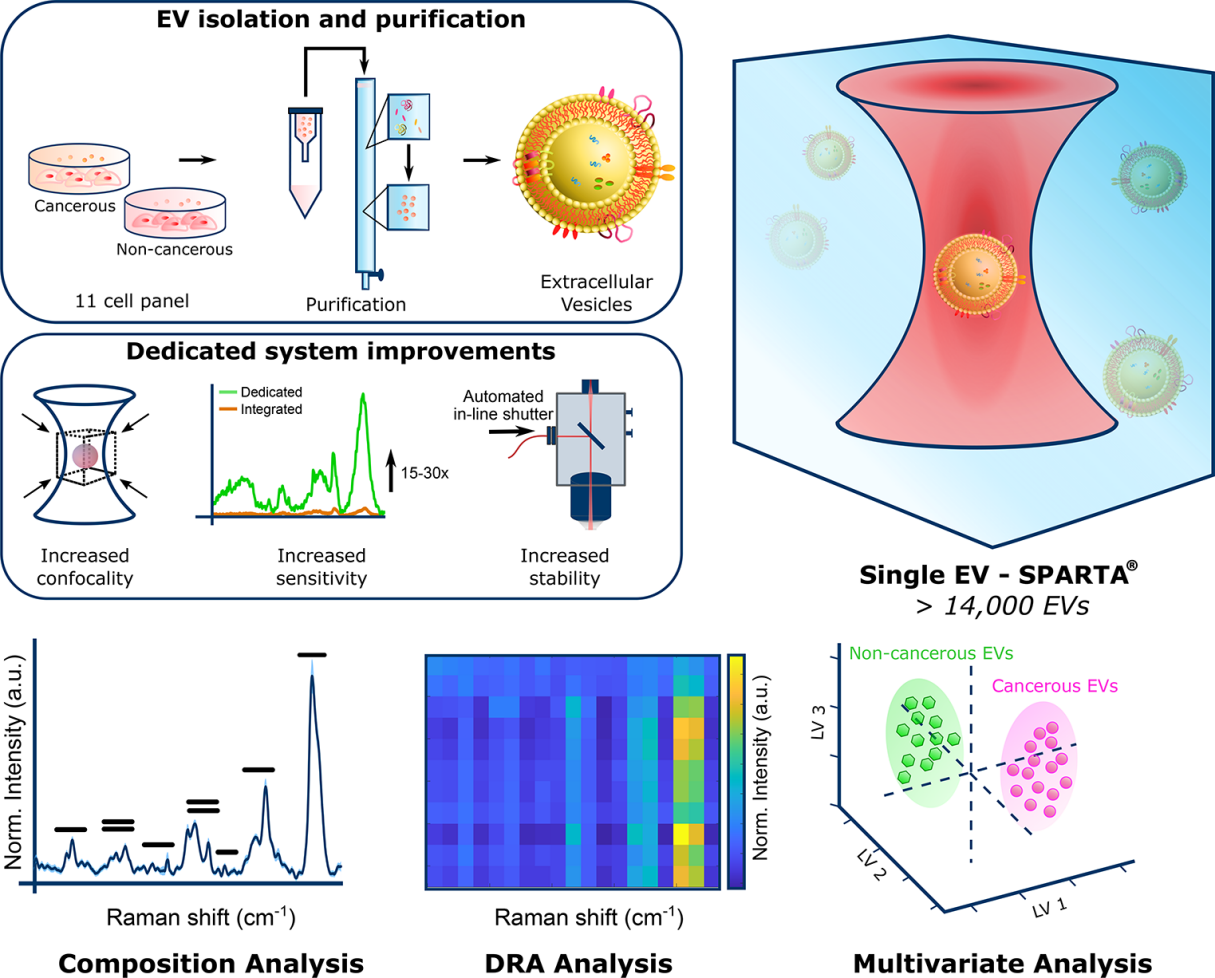
EVs as cancer biomarkers using the SPARTA system. EVs were isolated using stringently optimized methods from a panel of 11 breast cell lines (2 noncancerous, 9 cancer) and analyzed by SPARTA to obtain detailed compositional Raman spectra for over 14 000 individual EVs analyzed. Key improvements to our previously published method^45^ were made to achieve a dedicated automated high-throughput EV analysis platform with increased confocality, sensitivity, and stability. The obtained individual Raman spectra enable a detailed compositional assessment. Analyses by dimensional reduction arrays (DRAs) allow for the facile visualization and comparison of the large data sets. Multivariate analytical modeling allows for distinguishing the EV origin as noncancerous or cancer cell-derived with high sensitivity and specificity.

**Figure 2:**
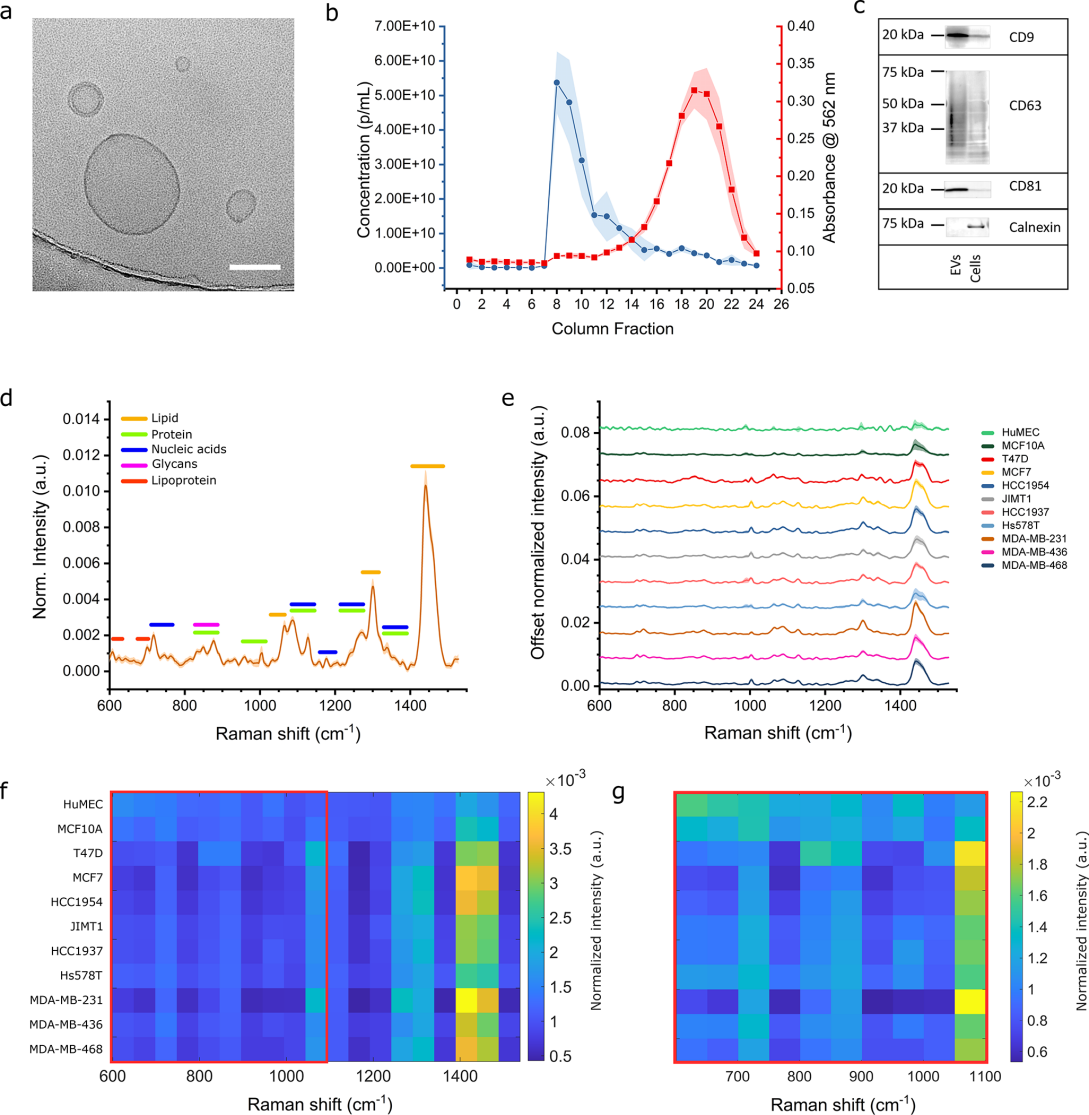
Comprehensive EV analysis. (a) Cryo-TEM image of purified MDA-MB-231-derived EVs (scale bar = 100 nm). (b) BCA protein quantification (*N* = 3, *n* = 3, mean ± s.d., red) and NTA particle analysis (*N* = 3, *n* = 5, mean ± s.d., blue) of SEC column trace fractions on the concentrated conditioned medium from MDA-MB-231 cells. (c) Western blot analysis for the EV markers CD9, CD63, and CD81 and the endoplasmic reticulum protein calnexin on MDA-MB-231 cells and purified EVs. (d) SPARTA Raman spectra compositional analysis of MDA-MB-231 breast cancer EVs (*n* = 320, mean ± s.d.), spectral regions pertaining to various signals of biological origin indicated as shown; see Table 2 for peak assignments. Comparison of the 11 EV panel (e) by stacked spectra (*n* > 223, mean ± s.d.) and (f) by dimensional reduction array (DRA), plotting the Raman intensities in 50 cm^–1^ bins on a heatmap for facile comparison. (g) Close-up analysis of the indicated red region in panel f. Spectra were all acquired using an acquisition time of 20 s.

We have designed a dedicated approach for the comprehensive analysis of EVs as potential cancer biomarkers, demonstrated here by analyzing cancerous and noncancerous breast cell-derived EVs. Several key factors exemplify this approach and address crucial limitations in the field, such as ensuring EV isolate purity and performing a sensitive, automated, and high-throughput analysis of unmodified EVs in their native state. As a result, a label-free, nondestructive analysis of EVs in their hydrated state is performed to identify the compositional differences between EVs of cancerous and noncancerous origin in an unbiased manner. An overview of our approach can be seen in Figure 1. Extensive research has been performed on optimizing EV isolation procedures to maximize the yield while ensuring high purity by excluding soluble factors.^48^ The combination of the ultrafiltration and size exclusion chromatography (SEC) steps performed here achieves high EV yield and purity, corroborated by column trace protein quantification, nanoparticle tracking analysis (NTA), Western and immunoblotting, and cryo-transmission electron microscopy (TEM).^48,49^ A comprehensive overview of the analysis of MDA-MB-231 EVs is shown in Figure 2a–c. Cryo-TEM confirms intact vesicles (Figure 2a), as we have also shown extensively in a recent study.^49^ NTA and BCA protein quantification measurements on the SEC column fractions confirm clear separation of the EV fractions with high particle concentration from the main soluble protein peak (Figure 2b). A small secondary protein peak corresponding to the EVs is shown in the close-up in Figure S1a. Immunoblotting (Dot blot) was also performed on the column fractions to corroborate NTA and protein quantification results, with the quintessential EV membrane protein markers CD9, CD63, and CD81 most strongly present in the same EV-containing fractions (Figure S1b). Figure 2c shows Western blotting performed to compare the expression of EV membrane protein markers and the endoplasmic reticulum protein calnexin on EVs versus their parent cells, showing significantly stronger expression of the EV markers in the EV isolate compared to the cell lysates, with the inverse holding true for calnexin expression. Western blots of EVs from the selected breast cell panel were performed to confirm expression of the EV markers for all cell types in this study (Table 1), showing their presence to various degrees (Figure S1c).

**Table 1:** Breast Cell Panel: Disease State and Receptor Expression of the 11 Cell Panel from Which EVs Were Isolated

cell line	disease state	receptor expression
HuMEC	noncancerous, primary	
MCF10A	noncancerous immortalized	
T47D	metastatic carcinoma	ER+
MCF7	metastatic carcinoma	ER+
HCC1954	primary carcinoma	HER2+
JIMT1	metastatic carcinoma	HER2+
HCC1937	primary carcinoma	triple negative
Hs578T	primary carcinoma	triple negative
MDA-MB-231	metastatic carcinoma	triple negative
MDA-MB-436	metastatic carcinoma	triple negative
MDA-MB-468	metastatic carcinoma	triple negative

After confirming that our purification procedure can isolate EVs with high purity, EVs were isolated in two biological replicates from each cell type in the panel and analyzed by NTA (Table S1). The resulting EV isolates were analyzed using the dedicated SPARTA system. Our SPARTA technology is ideally suited for analyzing EVs as it is designed to perform label-free, high-throughput analyses of single particles in their native state in a nondestructive manner. The previously established base technology^45^ was redesigned into a dedicated custom instrument to achieve key improvements in sensitivity and stability, as well as reducing the confocal volume to optimize trapping of EVs, most of which are in the 50–200 nm size range.

We applied this methodology to the comprehensive study of EVs derived from the aforementioned *in vitro* culture of breast epithelial cells, both primary and cell lines encompassing two noncancerous strains and nine breast cancer cell lines. The 11 cell type panel represents the relevant heterogeneity in disease state, *i.e.*, metastatic and nonmetastatic and differences in receptor expression, including cell lines that are ER+, HER2+, and triple negative, as summarized in Table 1. In total, over 14 000 single EV spectra were acquired over two isolations. The influence of the acquisition time per EV was investigated by varying from 10 to 20 s, to investigate if this would significantly affect the achievable sensitivity and specificity in distinguishing between the origin of the EVs.

The Raman spectra obtained through SPARTA contain a detailed fingerprint of the composition of the analyzed EVs, as exemplified by the spectrum (mean ± s.d., *n* = 320) in Figure 2d of MDA-MB-231 cell-derived EVs, a metastatic triple negative carcinoma. The colored bars indicate the peaks pertaining to the presence of lipids, proteins, glycans, nucleic acids, and lipoproteins. A detailed peak assignment of the compounds and vibrations present, based on the literature, is shown in Table 2. The compositional profile displayed in Figure 2d is exemplary of an EV, being a lipid vesicle containing an abundance of proteins and nucleic acids. The SPARTA approach characterizes the subtle changes in this fingerprint across all EVs within the cell panel to identify signatures that could indicate their cancerous or noncancerous origin and investigate the difference in cancer subtypes. A common and straightforward method of visualizing differences in the mean spectra is an overlay or stack plot, as shown in Figure 2e for all EV types tested here. However, comparing large data sets makes the identification of spectral differences difficult using overlay or stack representations. We therefore developed an alternative approach inspired by gene expression arrays where large sets of genes can be easily compared at a glance for up- and downregulation using color representations.^51,52^ The resulting dimensional reduction array (DRA) analysis is performed using custom MATLAB scripts and setting a user-specified bin size (in wavenumbers) for dimensional reduction and plotting the mean intensity for each bin on a color scale to create a heatmap of the Raman spectra, as shown in Figure 2f. It should be noted that the choice of bin size is important, as it directly impacts the resulting visualization and may cause peaks to be split over multiple bins if the bin size is too small. The dimensional mismatch between the CCD collection axis of the spectrograph and the true Raman shift axis in wavenumbers is automatically corrected to account for variations in data points per bin along with the Raman shift range. At a glance, the differences between the mean spectra of the EVs of the 11 cell type panel can be easily identified, such as a markedly higher lipid content of the cancer-derived EVs, compared to the noncancerous controls. The color gradient effectively indicates the relative differences in composition between the cancer subtypes.

**Table 2:** EV Composition Raman Peak Assignment: Peaks Assigned Based on the Compendium Tabulated by Movasaghi *et al.*(50)

band	vibration/compound	Raman shift (cm^–1^)
lipoprotein	cholesterol	609
lipoprotein	cholesterol	698
NA	DNA ring breathing modes	723
protein/glycans	tyrosine, glycans	845
protein/glycans	tyrosine, glycans	884
proteins	C—C stretch protein β-sheet	989
proteins	phenylalanine	1004
lipids	C—C stretch lipids	1064
proteins/NA	C—N stretch proteins, PO_2_ stretch DNA backbone	1084
proteins/NA	C—N stretch proteins	1128
NA	cytosine, guanine	1185
proteins/NA	amide-III proteins, DNA ring breathing modes	1256
lipids	CH_2_ twist lipids	1295
proteins/NA	amide-III proteins, DNA ring breathing modes	1337
proteins/NA	guanine, tryptophan	1354
proteins/NA	DNA ring breathing modes	1371
lipids	C=O stretch, CH_2_ lipids	1399
lipids	CH_2_ bend lipids	1442

A critical factor in the design of the DRA approach is the choice of a perceptually uniform color gradient. A commonly used “rainbow” gradient highlights relative differences stronger than the blue–green–orange–yellow (“parula”, MATLAB^53^) gradient used here, but it is not a perceptually uniform gradient.^53−55^ Since the color scale in the DRA is determined by the minimum and maximum values present, the construction of additional DRAs based on subsections of the Raman spectrum can be informative, as shown for the lower fingerprint range in Figure 2g, highlighting variations in nucleic acid and protein contributions to the spectra by avoiding the domination of the scale by the strong CH_2_-bend vibration at 1442 cm^–1^ originating from the presence of lipids. In addition to comparing means between many sets or classes, DRA analyses can be used to capitalize on the ability of SPARTA for single EV spectral acquisition, thus visualizing the intracell type spectral variation. The DRA analysis, therefore, provides an efficient and effective way of visualizing the large number of spectra acquired, with a total of over 3700 spectra visualized across 11 cell types in Figure S2. Overall, these plots show a relatively uniform representation within the cell types, as shown by the smooth resultant vertical color bands. More intrabatch variation is observed for the non-cancerous-derived EVs, and increased heterogeneity can be seen within the cancer sets for Hs578T-derived EVs in particular. This intrabatch variation is an important consideration for downstream classification approaches, as such approaches depend on the interbatch variation being larger than the intrabatch variation.

**Figure 3:**
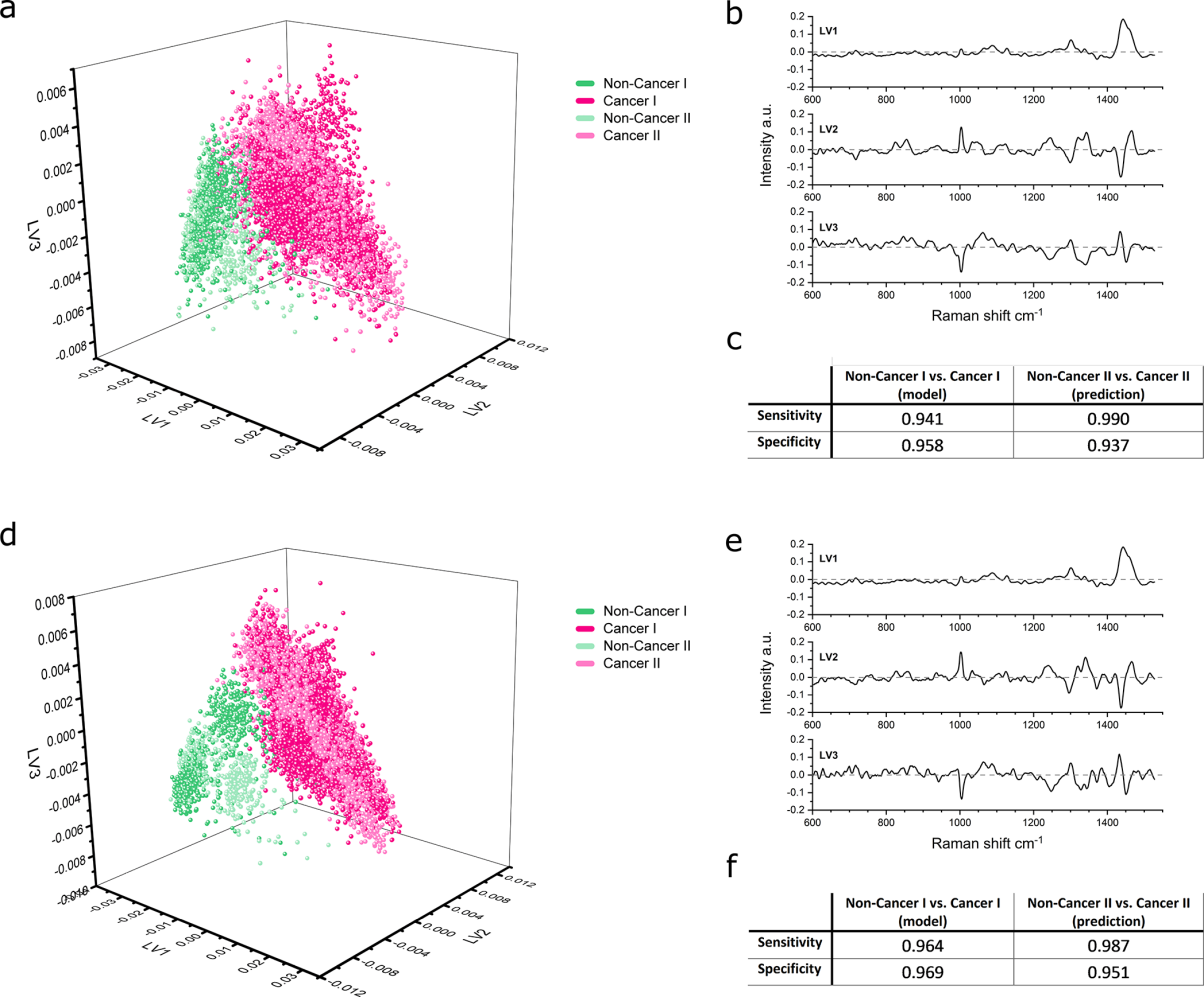
PLSDA modeling and predictive classification of cancer- and non-cancer-derived EVs. Modeling based on isolation I and applied to predict isolation II with 10 s acquisitions (a–c) and 20 s acquisitions (d–f). (a) 10 s acquisitions PLSDA score plot for LV1 against LV2 and LV3. Noncancer I (*n* = 671), Cancer I (*n* = 3558), Noncancer II (*n* = 599), and Cancer II (*n* = 2764) (*n* = 7592 total, 7558 included, outliers excluded based on Q residuals and Hotelling *T*^2^). (b) LV pseudospectra of the PLSDA model (LV1 63.96%, LV2 4.78%, LV3 2.59% variance captured). (c) PLSDA model cross-validated sensitivity and specificity table for the model and prediction, noncancer vs cancer. (d) 20 s acquisitions PLSDA score plot for LV1 against LV2 and LV3. Noncancer I (*n* = 509), Cancer I (*n* = 3273), Noncancer II (*n* = 401), and Cancer II (*n* = 2314) (*n* = 6497 total, 6456 included, outliers excluded based on Q residuals and Hotelling *T*^2^). (e) LV pseudospectra of the PLSDA model (LV1 66.75%, LV2 5.64%, LV3 3.03% variance captured). (f) PLSDA model cross-validated sensitivity and specificity table for the model and prediction, noncancer vs cancer.

Our primary aim was to use the large data sets acquired from the breast cell panel EVs to determine if Raman spectral analysis by SPARTA can accurately distinguish breast cancer cell-derived EVs from their noncancerous cell-derived counterparts. To investigate this capability, a multivariate statistical analysis in the form of partial least squares discriminant analysis (PLSDA) was utilized. PLSDA modeling is an effective dimension reductive approach which condenses the variance within the spectra into latent variables (LVs), with each sample assigned a score based on the relative contribution of each latent variable to describe its composition. PLSDA is a supervised method, using the class information to maximize classification separation, and was internally cross-validated in this instance using venetian blinds (10 splits). PLSDA models have been extensively and successfully applied to Raman-based classification studies, particularly in cancer.^56−59^ Here, we used the Raman spectra acquired from the first isolation of EVs from all cell types to build the PLSDA model and used it to independently validate the prediction performance of a secondary isolation of EVs. To answer the core question relating to cancer/noncancer classification, we pooled the data for the relevant sets together. We also measured all samples at 10 and 20 s high signal-to-noise ratio acquisition times with SPARTA, to investigate if longer acquisitions yielded a significantly higher classification performance. An overview of these results can be seen in Figure 3. The 10 s acquisition PLSDA model in Figure 3a shows a clear distinction between EVs derived from cancerous (red) and noncancerous (green) cells, shown by the separation between the clusters. A model was constructed based on the first isolation combining 671 spectra from HuMEC- and MCF10A-derived (noncancer) EVs and 3558 from the remaining cancer cell-derived EVs. The 3 LVs describing this model with the loadings or pseudospectra, as shown in Figure 3b, demonstrate the compositional variances between cancer- and non-cancer-derived EVs. LV1 as the core classifier is dominated by the strong positive lipid-associated peak at 1442 cm^–1^. The overall positive scoring on LV1 of the cancer-derived EVs and converse negative scoring of the noncancerous cell-derived EVs demonstrate, as indicated by the DRA analysis, the strong relative increase in lipid content of cancer cell-derived EVs. The model resulted in an excellent internally cross-validated sensitivity and specificity of 94.1% and 95.8%, respectively. Using this model to independently predict the classification of the EVs from a separate secondary isolation (*n* = 3363 combined spectra) provided an excellent prediction model achieving 99% sensitivity and 93.7% specificity (Figure 3c). The same multivariate modeling was performed on the separate sets of spectra acquired for each sample for both isolations at a high signal-to-noise ratio acquisition time of 20 s. As seen in Figure 3d–f, a similar model was obtained (noncancer *n* = 509, cancer *n* = 3273), confirming the effectiveness of capturing the intrasample variance.

Both the model and the prediction of the classification using the independent secondary isolation measurements achieved sensitivities and specificities of >95%. Surprisingly, the sensitivity and specificity were not markedly improved by doubling the spectral acquisition time, with only a very modest increase, mostly in specificity, observed for the model and prediction. This shows that 10 s acquisitions are sufficient to capture all the variance necessary for a very accurate cancer/noncancer classification of the EVs by SPARTA.

**Figure 4:**
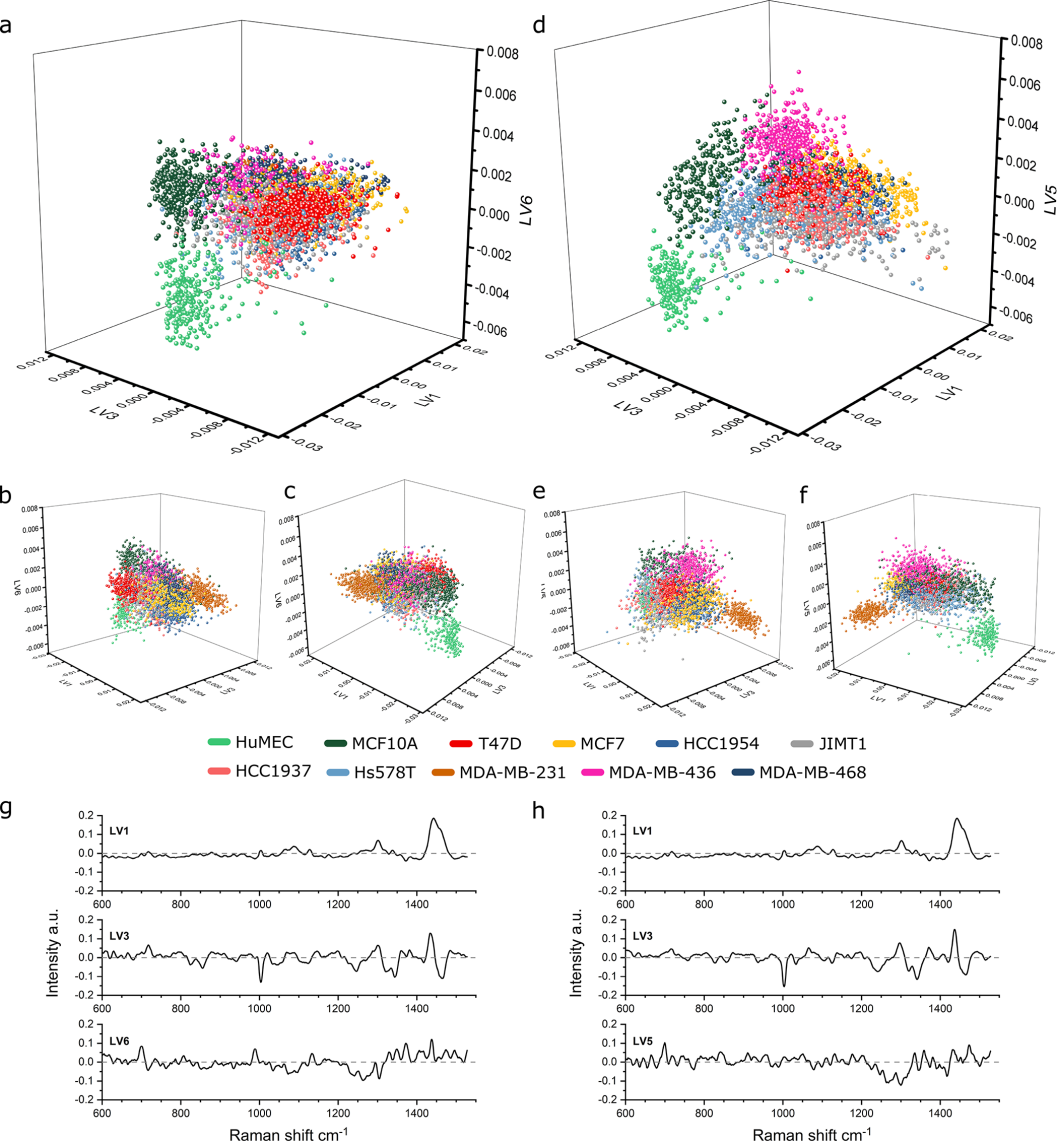
PLSDA modeling of 11 cell panel derived-EVs. (a) PLSDA score plot for a panel of 11 EVs at 10 s acquisitions plotted LV1 against LV3 and LV6. HuMEC (*n* = 260), MCF10A (*n* = 411), T47D (*n* = 381), MCF7 (*n* = 637), HCC1954 (*n* = 531), JIMT1 (*n* = 417), HCC1937 (*n* = 296), Hs578T (*n* = 129), MDA-MB-231 (*n* = 488), MDA-MB-436 (*n* = 301), and MDA-MB-468 (*n* = 378) (*n* = 4229 total, 4202 included in the model, outliers excluded based on Q residuals and Hotelling *T*^2^) with additional projections (b, c). (d) PLSDA score plot for a panel of 11 EVs at 20 s acquisitions plotted LV1 against LV3 and LV5. HuMEC (*n* = 242), MCF10A (*n* = 267), T47D (*n* = 371), MCF7 (*n* = 356), HCC1954 (*n* = 385), JIMT1 (*n* = 416), HCC1937 (*n* = 486), Hs578T (*n* = 325), MDA-MB-231 (*n* = 320), MDA-MB-436 (*n* = 391), and MDA-MB-468 (*n* = 223) (*n* = 3782 total, 3754 included in the model, outliers excluded based on Q residuals and Hotelling *T*^2^) with additional projections (e, f). (g) LV pseudospectra of the PLSDA model in panel a (LV1 64.08%, LV3 5.34%, LV6 0.78% variance captured). (h) LV pseudospectra of the PLSDA model in panel d (LV1 67.1%, LV3 6.60%, LV5 1.31% variance captured).

Following the successful classification of cancer cell- and noncancerous cell-derived EVs, we performed further modeling to investigate if it was possible to distinguish between the EVs derived from the 11 breast cell types, despite their closely related biological origin. Internally cross-validated PLSDA models were made for both 10 and 20 s acquisitions based on the first isolation, assigning each cell type its known class, as shown in Figure 4. For both models, 7 LVs were included, which balanced the minimization of the classification error against the potential for overfitting. Figure 4a–c shows the model based on 10 s acquisitions with three projections to visually assess the distinction between classes. Since a 3D plot is restricted to 3 LVs, these LVs were chosen based on their capability to visually distinguish as many different classes as possible, not necessarily those capturing the most overall variance. This is visualized in Figure S3 by plotting the scores on the relevant LV for each class (scatter plots and violin plots as shown). The clusters for the EVs derived from noncancerous cell types, HuMEC and MCF10A, can be easily distinguished from each other as well as from those derived from cancer cell types. Distinct clusters can be seen for most individual cancer cell types, limited by the visualization of only 3 of the 7 LVs. Using the 20 s acquisitions, a similar model is obtained as visualized in Figure 4d–f with the corresponding visualized LV score plots shown in Figure S4.

PLSDA model loadings, as shown for the 10 and 20 s acquisition models in Figure 4g,h, respectively, indicate the relative lipid signal as the core distinguishing factor between the EV sets, as for the models in Figure 3. For scoring, LVs containing more subtle variations in protein- and nucleic acid-related signals are also strong classifiers, particularly for the cancer subtypes.

The relative cross-validated sensitivities and specificities for EVs derived from each of the 11 cell types investigated are tabulated in Table 3. The corresponding receiver operated characteristic (ROC) curves are shown in Figures S5 and S6 for the 10 and 20 s models, respectively. Overall, very high classification sensitivity and specificity values were obtained, with near-perfect separation between the noncancerous breast epithelial cells (HuMEC and MCF10A) and all cancer subtypes. The total average areas under the curve for the ROCs were 0.925 and 0.947 for the 10 and 20 s models, respectively, a modest increase for the 20 s model. This is also reflected in Table 3, where the differences are small between the 10 and 20 s models. The largest gain is seen for the Hs578T-derived EVs, which may be attributed to the relatively large intrasample variance as seen on the DRA analysis (Figure S2), suggesting that longer acquisition times could be required for Hs578T-derived EVs.

**Table 3:** PLSDA Models Sensitivities and Specificities: Cross-Validated Calculated Sensitivity and Specificity for Each Cell Line in the Panel at 10 and 20 s Acquisitions per EV Spectrum, Models Shown in Figure 4a,d, Respectively

	10 s acquisitions		20 s acquisitions	
cell line	sensitivity	specificity	sensitivity	specificity
HuMEC	1.000	0.998	1.000	1.000
MCF10A	0.932	0.932	0.955	0.927
T47D	1.000	0.999	1.000	0.998
MCF7	0.889	0.837	0.862	0.757
HCC1954	0.949	0.739	0.890	0.770
JIMT1	0.858	0.808	0.897	0.881
HCC1937	0.858	0.700	0.716	0.803
Hs578T	0.637	0.687	0.847	0.883
MDA-MB-231	0.977	0.985	0.991	0.994
MDA-MB-436	0.903	0.829	0.961	0.929
MDA-MB-468	0.910	0.845	0.941	0.792

Given that the clustering of EVs largely corresponded to the individual cancer cell types from the PLSDA models, we decided to explore whether we could identify intrinsic biomolecular differences between the EVs derived from cancerous cell lines in comparison to those of noncancerous cells, for a deeper understanding of the factors driving clustering and demarcation of cancer EVs at a biomolecular level. However, in Raman trapping analysis, spatial dimensions are in principle singleton dimensions, and acquired spectra are a mixture of all biomolecular species present in the focal volume at the time of trapping. Therefore, spectral deconvolution techniques that rely on spatial heterogeneity of the sample to identify “pure” biomolecular species, such as simplex maximization techniques, are generally not applicable as they would describe all EV samples based on a few EVs at the hyperspectral extremes. Instead, we identified spectral bands attributed to key biomolecular species, such as nucleic acids, sphingolipids, and protein (Figure S7a), with the aim of recovering spectral signatures of “pure” biomolecular species, without imposing spectral priors on the unmixing process. Through band-target entropy minimization,^60^ we recovered spectra indicative of phosphatidylcholine- and sphingomyelin-like species, protein, and nucleic acids for 10 and 20 s integration times (Figure S7b,c). The band-target entropy minimization algorithm belongs to the class of self-modeling curve resolution techniques and has been found particularly well-suited for recovering analytes only present in trace amounts compared to existing techniques. Since the recovered spectra, just as the input EV spectra, display intensity values as a function of Raman shift, prominent peaks can be readily assigned (Table S2), indicating the major Raman spectral bands present in these compounds.

**Figure 5:**
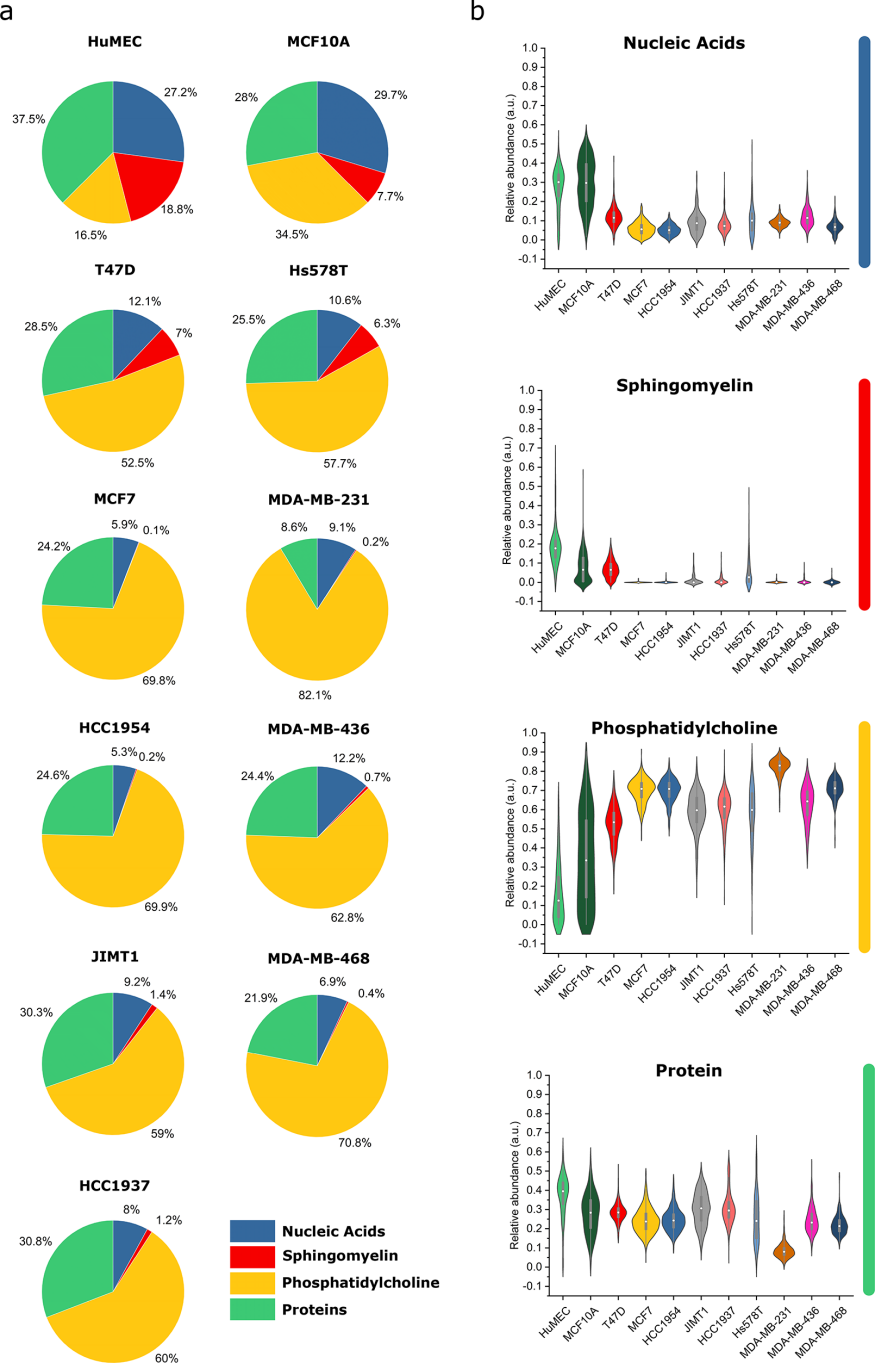
Entropy minimization composition analysis of cancer- and non-cancer-derived EVs (10 s acquisitions). (a) Pie charts indicating the relative abundance of identified components. (b) Violin plots for each component show the distribution between cancer and noncancer EV subtypes with the median indicated by a white point and 1.5 interquartile range.

Using the spectra of the recovered species, we calculated relative abundances by convex mixing for each trapped EV, to obtain a compositional distribution for each EV population at both 10 and 20 s integration times (Figure 5a,b and Figure S8a,b, respectively). Most cells in the panel appeared to secrete EV populations with unique compositional distributions. A striking difference can be seen for the extracted average compositional profiles of the noncancer cell-derived EVs (HuMEC and MCF10A) compared to those of the cancer cell-derived ones. HuMEC- and MCF10A-derived EVs show a markedly higher relative nucleic acid content and a proportionally lower lipid content, as indicated by the phosphatidyl choline extracted component. This clear differentiation in composition between cancer and noncancer cell-derived EVs confirms the biological basis for the high classification sensitivity and specificity as obtained by the PLSDA analyses. Looking closer at differences between cancer cell line-derived EVs, we found similar compositions at 10 s integration time for MCF7- and HCC1954- and for JIMT1- and HCC1937-derived EVs (Figure 5a). Interestingly, the MCF7–HCC1954 pair was also associated with significant misclassification based on clustering using 7 LVs from the PLSDA models. The biggest difference between EVs from the two cancer lines was in the nucleic acid content, which differed only by <1% point on average between the two groups (Figure 5a). The same is true for JIMT1 and HCC1937 EVs, with the largest difference a 1.2% point change in the nucleic acid content. For 20 s integration time, differences in the recovered spectra were seen for protein and sphingomyelin-like species, particularly around the phenylalanine peak at 1003 cm^–1^, and at 989 cm^–1^ for sphingomyelin-like species compared to the recovered spectra at 10 s integration time (Figure S7b,c). Relative abundances calculated assuming convex mixing indicated less presence of sphingomyelin-like species in the EVs, compared to that observed at 10 s integration time (Figure S8a,b). Even so, compositions among MCF7, HCC1954, and MDA-MB-468 remained similar to those observed at 10 s integration time, as did their relative misclassification in the PLSDA modeling. Overall, these results suggest that a minimum 2% point difference in relative abundances for at least one of the components as resolved by the current entropy minimization analysis is required to achieve good separation by PLSDA modeling. The above results demonstrate the powerful combination of PLSDA-type modeling and entropy minimization techniques for the characterization of the EV signatures as derived by SPARTA. Relating the biomolecular compositions of the EVs to the receptor expressions of the parent cells (Table 1), no clear trend was observed. This indicates that the differences in cellular receptor expression do not directly translate into conserved changes in the overall biomolecular makeup of the EVs, which supports the complementary nature of the agnostic-type approach of SPARTA-based EV analysis.

Crucially, reference spectra were not used to recover biomolecular spectra or calculate the relative biomolecular compositions. Recovered spectra were reconstructed based on the minimization of entropy around selected band-targets, resulting in spectra indicative of protein, nucleic acids, and sphingomyelin-like and phosphatidylcholine-like species, all of which are known constituents of extracellular vesicles.^18,61^ However, due care must be paid to the interpretation of the relative abundances and the compositional analysis. While we were able to recover four key biomolecular species of the EV composition, there are undoubtedly other less pronounced constituents that were not recovered, which ultimately may affect the relative abundances calculated assuming convex mixing. Depending on the data set, peaks considered interferents of particular biomolecular species may occur in the recovered spectra of another distinct biomolecular species, which in turn affects the following compositional analysis. This appears to be the case for sphingomyelin-like species between the 10 and 20 s integration times. However, to our knowledge, direct, label-free *in vitro* characterization of EVs at a biomolecular level has not previously been demonstrated through spectral deconvolution without using a reference library. Here, we have shown the prospects of exploiting the high-throughput nature of SPARTA particle trapping and the information-rich nature of Raman spectral data for direct compositional analysis, which we believe exhibits exciting potential both for fundamental EV research and, ultimately, for the development of diagnostic techniques.

## Conclusions

This study advances the state-of-the-art through the use of stringent EV isolation protocols that limit the coprecipitation of contaminants in the EV isolate, the use of appropriate noncancerous cell controls that show that our system can differentiate EVs of cancerous origin, and the application of our dedicated SPARTA system for the characterization of single EVs in a high-throughput manner. Our approach exhibits promising clinical diagnostic utility and research utility as SPARTA is capable of effectively characterizing and comparing the composition of closely related EV subsets.

The application of SPARTA in the clinical setting faces several challenges. First, our study performs a comprehensive analysis of EVs derived from *in vitro* cell culture, which acts as an appropriate precursor to analyses of EVs derived from healthy volunteers and patients suffering from breast cancer. Data derived from human liquid biopsies is critical to fully explore the clinical utility of this promising diagnostic modality. Future studies are planned to analyze EVs derived from the plasma of human subjects using the methods outlined. Second, a relatively high concentration of EVs is required to achieve reliable trapping using the SPARTA system, and several sample purification and concentration steps are still necessary prior to measurement. Future studies will focus on modifications to the system that can effectively trap with lower concentrations of EVs and ensure that clinically relevant concentrations of EVs isolated from patients can be reliably analyzed, with minimal sample processing requirements.

Our findings bring EV-based diagnostics of cancer closer to the clinic by virtue of our dedicated SPARTA system, which is capable of high-throughput analysis of single EVs in their native state. We demonstrated the key benefits of Raman-based single particle analysis for EVs, both as a tool to study the fundamental biological intricacies of EVs through profiling changes in their composition as well as for future diagnostic utility. Our approach is not limited to breast cancer, as EVs are released from almost all cell types, and their role in multiple cancers is well-established. Establishing liquid biopsies as a reliable standard of care in cancer diagnostics could enable routine monitoring of cancer treatment efficacy through a minimally invasive sampling of biofluids. Based on the positive findings of this study, we feel that our dedicated SPARTA system warrants further validation using patient-derived EVs prior to deploying the standalone system as a versatile diagnostic tool for minimally invasive cancer identification.

## Methods

### Dedicated SPARTA System

A fully custom and dedicated SPARTA system was built based on our previously reported commercial-based setup (alpha300R+, WITec, Ulm, Germany).^45^ This custom SPARTA system was fully optimized for the analysis of EVs. As EVs are highly complex vesicles, both in composition and in intrinsic biological variability, a high number of traps and samples are required for their comprehensive analysis. Therefore, a number of factors were identified which could be improved upon to design a dedicated SPARTA system, including laser power and stability, spectral range, and confocality.

The basic microscope setup was built with the Cerna platform (Thorlabs). A spectrograph (HoloSpec-F/1.8-NIR, Andor, UK) was coupled with an iDus 416A-LDC-DD (Andor) thermoelectrically cooled (−60 °C) back-illuminated CCD camera. For optical trapping and simultaneous Raman excitation, a 200 mW 785 nm laser (Cheetah, Sacher Laser Technik) was used, temperature-controlled through a laser diode control box (PilotPZ 0500, Sacher Laser Technik). A 50 μm optical fiber was used as the confocal pinhole to collect the scattered light. Measurements were conducted at 375 mA laser diode current, resulting in 135 mW of output power before the objective and 100 mW after the objective (focus). An in-line shutter (SHB05T, Thorlabs) and controller were used to enable and disable the optical trap when required. The samples were interfaced with a 63× 1.0 NA water immersion objective (W Plan Apochromat, Zeiss). The sample slide was supported by a Zaber automated lift-stage (X-VSR40A, Zaber Technologies Inc.) controlled through a custom joystick button controller (Laser 2000). The spectrograph, camera, shutter, and stage were controlled through custom MATLAB 2016b scripts (MathWorks).

### Cell Culture and EV Isolation

A breast cancer cell panel was compiled consisting of MCF7, HCC1937, HCC1964, Hs578T, JIMT1, MDA-MB-231, MDA-MB-436, MDA-MB-468, and T47D cancer cells and the noncancerous HuMEC and MCF10A cells. All except HuMEC were subjected to STR profiling for authentication. The disease state and receptor status of each cell type are detailed in Table 1. Cells were cultured to confluence at 37 °C and 5% CO_2_.

HuMEC cells were cultured in HuMEC ready medium [HuMEC basal serum-free medium (12753018)] supplemented with a HuMEC supplement kit (12755013, Thermo-Fischer). MCF10A cells were cultured in DMEM/F12 media supplemented with 5% (v/v) horse serum (Invitrogen), 20 ng/mL EGF (Peprotech), 0.5 μg/mL hydrocortisone (Sigma), 100 ng/mL cholera toxin (Sigma), 10 μg/mL insulin (Sigma), and 1× penicillin/streptomycin (Gibco). All other cell lines were cultured in high-glucose DMEM supplemented with 10% (v/v) FBS, 20 mM HEPES, 1× nonessential amino acids (NEAA, Gibco), and 1× penicillin/streptomycin (Gibco), with media changes every 2 days.

For EV isolation, cells were cultured to ∼90% confluence, after which cell culture media were changed for serum-free equivalents. Cells were cultured for an additional 3 days, after which the conditioned medium was collected and centrifuged at 300 rcf for 5 min, and the supernatant was passed through a bottle top filter with a 0.45 μm pore size (VWR). The media was concentrated to approximately 500 μL by ultrafiltration (Amicon Ultra-15, 100 kDa) and further purified by size exclusion chromatography over a Sepharose CL-2B (Sigma-Aldrich) column of 30 × 1 cm. 1 mL fractions were collected, and the EV containing fractions, as verified by nanoparticle tracking analysis (NTA) (Malvern) (typically fractions 8–12), were pooled. The results of NTA analysis and details regarding the concentration of EVs obtained from each cell line are included in Table S1. The EVs were stored frozen at −80 °C and kept in an ice bath prior to Raman spectroscopic analysis.

### Cryo-Transmission Electron Microscopy of EVs

Cryo-TEM samples were prepared using an automatic plunge freezer (Thermo Fisher/FEI Vitrobot). 3.5 μL of purified MDA-MB-231 EVs was spotted on glow-discharged (air, 1 min 30 mA) holey carbon copper grids with a thin continuous carbon film. The surplus of the sample was blotted onto filter paper. The resulting film was subsequently vitrified in liquid ethane. Samples were stored in liquid nitrogen and imaged at −179 °C (Gatan cryo-holder) in a JEOL 2100F transmission electron microscope. Micrographs were obtained at 200 kV in low electron dose mode.

### BCA Protein Quantification

The BCA protein assay for determining protein concentrations was performed using the BCA protein assay kit (Thermo Fischer Sci). 10 μL of each sample was used, to which 200 μL of BCA reagent, consisting of 49 parts A and 1 part B, was added. Samples were incubated at 37 °C for 30 min. Absorbance was measured at 562 nm on a Spectramax M5 instrument (Molecular Devices, San Jose, CA).

### Dot Blot Analysis

Dot blotting was performed using the BioDot apparatus (Bio-Rad) on a 0.45 μm nitrocellulose membrane (Bio-Rad). Membranes were prewetted in Tris-buffered saline (TBS), after which 100 μL of each column fraction was applied. Membrane blocking was performed for 1 h at room temperature in 5% (w/v) nonfat dry milk (Bio-Rad) in TBS with 0.1% (v/v) Tween-20 (Sigma). Three washes in TBS-T were performed for 10 min each. Membranes were incubated overnight at 4 °C in one of three primary antibodies: mouse-anti-CD9 (Thermo Fischer Sci, 10626D), mouse-anti-CD63 (Thermo Fischer Sci, 10628D), or mouse-anti-CD81 (Thermo Fischer Sci, 10630D), all diluted 1:1000 in 5% (w/v) bovine serum albumin (BSA, Sigma) in TBS-T. The negative control was incubated in 5% (w/v) BSA in TBS-T. Three washes in TBS-T were performed for 10 min each. Membranes were incubated for 1 h at room temperature with LiCor-dye 800 CW-conjugated goat-antimouse (LiCor Biosciences) secondary antibody diluted 1:10 000 in TBS-T. Three washes in TBS-T were performed for 10 min each. Membranes were imaged on a LiCor Odyssey imager (LiCor) and quantified using LiCor ImageStudio software.

### Western Blot Analysis

Cell and EV samples were lysed for protein isolation using RIPA buffer (Cell Signaling Technology, Danvers, MA), to which phosphatase and protease inhibitors (Roche, Basel, Switzerland) were added. Lysates were sonicated for 20 s at 20% amplitude on ice with a VibraCell VCX500 sonicator (Sonics & Materials Inc., Newtown, CT). Next, samples were gently mixed for 1 h at 4 °C. Samples were centrifuged at 4 °C, 20000*g* for 10 min and supernatants collected. 15 μg of protein was added to Laemmli sample buffer (Bio-Rad Laboratories, Inc., Hercules, CA) and separated by SDS-PAGE on Criterion XT Precast 4–12% Bis-Tris gels (Bio-Rad). Next, the protein was blotted onto PVDF membranes (EMD Millipore, Burlington, MA). Membranes were blocked in 5% (w/v) nonfat dry milk (Bio-Rad) in TBS with 0.1% (v/v) Tween-20 (Sigma) for 1 h at room temperature. Three washes in TBS-T were performed for 10 min each. Membranes were incubated overnight at 4 °C in one of four primary antibodies: mouse-anti-CD9 (Thermo Fischer Sci, 10626D), mouse-anti-CD63 (Thermo Fischer Sci, 10628D), mouse-anti-CD81 (Thermo Fischer Sci, 10630D), or rabbit-anticalnexin (2679, Cell Signaling Technology), all diluted 1:1000 in 5% (w/v) bovine serum albumin (BSA, Sigma) in TBS-T. Three washes in TBS-T were performed for 10 min each. Membranes were incubated for 1 h at room temperature with LiCor-dye 800 CW-conjugated goat-antimouse or goat-antirabbit (LiCor Biosciences) secondary antibody diluted 1:10 000 in TBS-T. Three washes in TBS-T were performed for 10 min each. Membranes were imaged on a LiCor Odyssey imager (LiCor) and quantified using LiCor ImageStudio software.

### Nanoparticle Tracking Analysis

The concentration of EVs was measured on a Nanosight NS300 instrument with a 532 nm laser and sCMOS camera (Malvern). Samples were diluted in particle-free DPBS, where possible to a concentration of 10^8^–10^9^ particles/mL. Using NTA V3.0 software, 3 30 s videos were recorded in different fields. The camera level was kept at 15, and the detection threshold for analysis was 5.

### SPARTA Analysis of EV Composition

EV samples isolated from the breast cancer panel were thawed on ice, and 240 μL of solution was placed on a 22 mm coverslip fixed on a standard microscopy slide. Using the dedicated SPARTA system, each EV sample was analyzed with an acquisition time of either 10 or 20 s over a 4 h period. After 4 h, sample evaporation made spectral acquisition impossible. For each cell isolation, two measurements were performed, one with 10 s and one with 20 s acquisition times, resulting in approximately 400–500 μL per isolation, per cell line. The spectra of the successfully trapped EVs were processed. A representative spectrum is shown in Figure 2a with the accompanying explanation of the peaks detailed in Table 2. The mean spectra ± s.d. were calculated in MATLAB 2019b and plotted in OriginPro 2020. PLSDA multivariate statistical modeling was performed using the PLS Toolbox 8.8.1 (Eigenvector Research Inc.).

### Spectral Processing

The Raman spectra were imported into MATLAB and processed using custom-made analysis scripts. Cosmic spikes were removed from the data based on peak amplitude and a threshold on the second derivate. A spectral response correction was applied based on the measurement of a relative intensity correction sample for 785 nm excitation as supplied by the NIST (National Institute of Standards and Technology, US, SRM2241).

Minimum and maximum postthresholding were conducted if applicable, by manual threshold decision to remove background solution spectra or spectra of clearly nonsingle particle origin (*e.g.*, aggregates). A primary background subtraction was performed by subtraction of 95% intensity of averaged spectra of PBS (*n* = 200–220, separate sets for isolation 1 and 2 and at 10 and 20 s acquisition times per spectrum, respectively), and the data was cropped to the fingerprint region of interest, followed by a Whittaker baseline subtraction. Smoothing of the spectra was conducted by applying a first-order Savitzky–Golay smoothing filter with a frame size of 7, and normalization of the data was applied where applicable by division by the area under the curve.

### Dimensional Reduction Array (DRA) Construction

Dimensional Reduction Arrays (DRAs) were constructed based on the processed, averaged Raman spectra of the breast cancer cell panel-derived EVs. A custom MATLAB script and function were made consisting of truncating the data to the desired spectral range. A set block size of 50 cm^–1^ chosen for dimensional reduction and the CCD positions of the data were matched with the correct wavenumber positions (Raman shift). The reduced spectra were plotted on a colormap based on the MATLAB “perula” preset for uniform intensity presentation.

### Hyperspectral Entropy Minimization

Hyperspectral unmixing by entropy minimization was carried out based on the first isolation of cancerous and noncancerous EVs for both 10 and 20 s integration times. First, all trapping spectra of EVs were pooled in two separate data matrices, one for each of the integration times. All preprocessing following pooling was done in batch on all data in each of the respective data matrices. Next, baselines were removed by a Whittaker filter using an asymmetry parameter of 10^–4^ and a smoothing parameter of 10^4^. Based on inspection of the singular vectors from a singular value decomposition of the data and mean spectra of each of the 11 EVs derived from cancerous and noncancerous cell lines, a series of highly overlapped candidate bands were identified for entropy minimization to recover the spectrum of the associated “pure” biomolecular species. The analysis was based on band-target entropy minimization,^60,62,63^ using 10 loading vectors for recovery of sphingomyelin-like, phosphatidylcholine-like, and protein species, and 25 loading vectors for the recovery of nucleic acids in the data. The selection of loading vectors was the same for both data matrices, *i.e*., for 10 and 20 s acquisitions. Search for a global minimum in the entropy-based objective function was done using Simulated Annealing from the MATLAB Global Optimization toolbox, running in MATLAB R2021a (MathWorks) by pseudorandom initialization. The search for a global minimum was repeated 10 times for each of the band-targets, and the recovery with the lowest entropy was reported. Recovered “pure” spectra and EV mixture spectra were vector normalized to unit-length, and weights of each of the recovered spectra were calculated assuming convex mixing, non-negativity sum-to-one constrained least-squares fitting.

## Supporting Information Available

Supplementary figures and tables on BCA protein quantification and SEC column trace fractions, Western blot analysis of EV markers, NTA data for each EV sample, dimensional reduction array analysis of isolation 1 (20 s acquisitions), PLSDA LV score plots (10 and 20 s acquisitions), ROC curves for the PLSDA models (10 and 20 s acquisitions), entropy minimization spectra and band assignments, and entropy minimization composition analysis (20 s acquisitions) (PDF)

## Accession Codes

Raw data and MATLAB code for DRA analysis are available upon reasonable request from rdm-enquiries@imperial.ac.uk.

## References

[ref1] IARC WHO. Global Cancer Observatory World Fact sheet. https://gco.iarc.fr/today/data/factsheets/populations/900-world-fact-sheets.pdf (accessed 2021-06-14).

[ref2] Polyak K. Heterogeneity in Breast Cancer. J. Clin. Invest. 2011, 121, 3786–3788. 10.1172/JCI60534. 21965334 PMC3195489

[ref3] Pusztai L.; Mazouni C.; Anderson K.; Wu Y.; Symmans W. F. Molecular Classification of Breast Cancer: Limitations and Potential. Oncologist 2006, 11 (8), 868–877. 10.1634/theoncologist.11-8-868. 16951390

[ref4] Keller M. D.; Vargis E.; de Matos Granja N.; Wilson R. H.; Mycek M.-A.; Kelley M. C.; Mahadevan-Jansen A. Development of a Spatially Offset Raman Spectroscopy Probe for Breast Tumor Surgical Margin Evaluation. J. Biomed. Opt. 2011, 16 (7), 077006 10.1117/1.3600708. 21806286 PMC3144975

[ref5] Thomas G.; Nguyen T. Q.; Pence I. J.; Caldwell B.; O’Connor M. E.; Giltnane J.; Sanders M. E.; Grau A.; Meszoely I.; Hooks M.; Kelley M. C.; Mahadevan-Jansen A. Evaluating Feasibility of an Automated 3-Dimensional Scanner Using Raman Spectroscopy for Intraoperative Breast Margin Assessment. Sci. Rep. 2017, 7 (1), 13548 10.1038/s41598-017-13237-y. 29051521 PMC5648832

[ref6] Ralbovsky N. M.; Lednev I. K. Raman Spectroscopy and Chemometrics: A Potential Universal Method for Diagnosing Cancer. Spectrochim. Acta, Part A 2019, 219, 463–487. 10.1016/j.saa.2019.04.067. 31075613

[ref7] Alfano R. R.; Liu C. H.; Sah L. W.; Zhu H. R.; Akins D. L.; Cleary J.; Prudente R.; Cellmer E. Human Breast Tissues Studied by IR Fourier Transform Raman Spectroscopy. In Lasers in the Life Sciences; Bufton Glass A., Hsu T., Krupke W. J., Eds.; OSA Technical Digest; Optical Society of America: Baltimore, MD, 1991; Vol. 4, pp 23–28.

[ref8] Haka A. S.; Shafer-Peltier K. E.; Fitzmaurice M.; Crowe J.; Dasari R. R.; Feld M. S. Diagnosing Breast Cancer by Using Raman Spectroscopy. Proc. Natl. Acad. Sci. U. S. A. 2005, 102 (35), 12371–12376. 10.1073/pnas.0501390102. 16116095 PMC1194905

[ref9] Stone N.; Baker R.; Rogers K.; Parker A. W.; Matousek P. Subsurface Probing of Calcifications with Spatially Offset Raman Spectroscopy (SORS): Future Possibilities for the Diagnosis of Breast Cancer. Analyst 2007, 132 (9), 899–905. 10.1039/b705029a. 17710265

[ref10] Lyng F. M.; Traynor D.; Nguyen T. N. Q.; Meade A. D.; Rakib F.; Al-Saady R.; Goormaghtigh E.; Al-Saad K.; Ali M. H. Discrimination of Breast Cancer from Benign Tumours Using Raman Spectroscopy. PLoS One 2019, 14 (2), e0212376 10.1371/journal.pone.0212376. 30763392 PMC6375635

[ref11] Lazaro-Pacheco D.; Shaaban A. M.; Rehman S.; Rehman I. Raman Spectroscopy of Breast Cancer. Appl. Spectrosc. Rev. 2020, 55 (6), 439–475. 10.1080/05704928.2019.1601105.

[ref12] Algar W. R.; Prasuhn D. E.; Stewart M. H.; Jennings T. L.; Blanco-Canosa J. B.; Dawson P. E.; Medintz I. L. The Controlled Display of Biomolecules on Nanoparticles: A Challenge Suited to Bioorthogonal Chemistry. Bioconjugate Chem. 2011, 22 (5), 825–858. 10.1021/bc200065z. 21585205

[ref13] Warner E.; Plewes D. B.; Hill K. A.; Causer P. A.; Zubovits J. T.; Jong R. A.; Cutrara M. R.; DeBoer G.; Yaffe M. J.; Messner S. J.; Meschino W. S.; Piron C. A.; Narod S. A. Surveillance of BRCA1 and BRCA2Mutation Carriers with Magnetic Resonance Imaging, Ultrasound, Mammography, and Clinical Breast Examination. J. Am. Med. Assoc. 2004, 292 (11), 1317–1325. 10.1001/jama.292.11.1317. 15367553

[ref14] Siravegna G.; Marsoni S.; Siena S.; Bardelli A. Integrating Liquid Biopsies into the Management of Cancer. Nat. Rev. Clin. Oncol. 2017, 14 (9), 531–548. 10.1038/nrclinonc.2017.14. 28252003

[ref15] Shin H.; Oh S.; Hong S.; Kang M.; Kang D.; Ji Y. G.; Choi B. H.; Kang K. W.; Jeong H.; Park Y.; Kim H. K.; Choi Y. Early-Stage Lung Cancer Diagnosis by Deep Learning-Based Spectroscopic Analysis of Circulating Exosomes. ACS Nano 2020, 14 (5), 5435–5444. 10.1021/acsnano.9b09119. 32286793

[ref16] Krebs M. G.; Metcalf R. L.; Carter L.; Brady G.; Blackhall F. H.; Dive C. Molecular Analysis of Circulating Tumour Cells - Biology and Biomarkers. Nat. Rev. Clin. Oncol. 2014, 11 (3), 129–144. 10.1038/nrclinonc.2013.253. 24445517

[ref17] Stoorvogel W.; Kleijmeer M. J.; Geuze H. J.; Raposo G. The Biogenesis and Functions of Exosomes. Traffic 2002, 3 (5), 321–330. 10.1034/j.1600-0854.2002.30502.x. 11967126

[ref18] Vlassov A. V.; Magdaleno S.; Setterquist R.; Conrad R. Exosomes: Current Knowledge of Their Composition, Biological Functions, and Diagnostic and Therapeutic Potentials.. Biochim. Biophys. Acta, Gen. Subj. 2012, 1820 (7), 940–948. 10.1016/j.bbagen.2012.03.017. 22503788

[ref19] El-Andaloussi S.; Lee Y.; Lakhal-Littleton S.; Li J.; Seow Y.; Gardiner C.; Alvarez-Erviti L.; Sargent I. L.; Wood M. J. A. Exosome-Mediated Delivery of SiRNA *in Vitro* and *in Vivo*. Nat. Protoc. 2012, 7 (12), 2112–2126. 10.1038/nprot.2012.131. 23154783

[ref20] Armstrong J. P. K.; Holme M. N.; Stevens M. M. Re-Engineering Extracellular Vesicles as Smart Nanoscale Therapeutics. ACS Nano 2017, 11 (1), 69–83. 10.1021/acsnano.6b07607. 28068069 PMC5604727

[ref21] Hoshino A.; Costa-Silva B.; Shen T. L.; Rodrigues G.; Hashimoto A.; Tesic Mark M.; Molina H.; Kohsaka S.; Di Giannatale A.; Ceder S.; Singh S.; Williams C.; Soplop N.; Uryu K.; Pharmer L.; King T.; Bojmar L.; Davies A. E.; Ararso Y.; Zhang T.; et al. Tumour Exosome Integrins Determine Organotropic Metastasis. Nature 2015, 527 (7578), 329–335. 10.1038/nature15756. 26524530 PMC4788391

[ref22] Costa-Silva B.; Aiello N. M.; Ocean A. J.; Singh S.; Zhang H.; Thakur B. K.; Becker A.; Hoshino A.; Mark M. T.; Molina H.; Xiang J.; Zhang T.; Theilen T. M.; García-Santos G.; Williams C.; Ararso Y.; Huang Y.; Rodrigues G.; Shen T. L.; Labori K. J.; et al. Pancreatic Cancer Exosomes Initiate Pre-Metastatic Niche Formation in the Liver. Nat. Cell Biol. 2015, 17 (6), 816–826. 10.1038/ncb3169. 25985394 PMC5769922

[ref23] Peinado H.; Alečković M.; Lavotshkin S.; Matei I.; Costa-Silva B.; Moreno-Bueno G.; Hergueta-Redondo M.; Williams C.; García-Santos G.; Ghajar C. M.; Nitadori-Hoshino A.; Hoffman C.; Badal K.; Garcia B. A.; Callahan M. K.; Yuan J.; Martins V. R.; Skog J.; Kaplan R. N.; Brady M. S.; et al. Melanoma Exosomes Educate Bone Marrow Progenitor Cells toward a Pro-Metastatic Phenotype through MET. Nat. Med. 2012, 18 (6), 883–891. 10.1038/nm.2753. 22635005 PMC3645291

[ref24] Krafft C.; Wilhelm K.; Eremin A.; Nestel S.; von Bubnoff N.; Schultze-Seemann W.; Popp J.; Nazarenko I. A Specific Spectral Signature of Serum and Plasma-Derived Extracellular Vesicles for Cancer Screening.. Nanomedicine 2017, 13 (3), 835–841. 10.1016/j.nano.2016.11.016. 27965168

[ref25] Smith Z. J.; Lee C.; Rojalin T.; Carney R. P.; Hazari S.; Knudson A.; Lam K.; Saari H.; Ibañez E. L.; Viitala T.; Laaksonen T.; Yliperttula M.; Wachsmann-Hogiu S. Single Exosome Study Reveals Subpopulations Distributed among Cell Lines with Variability Related to Membrane Content. J. Extracell. Vesicles 2015, 4 (1), 28533 10.3402/jev.v4.28533. 26649679 PMC4673914

[ref26] Carney R. P.; Hazari S.; Colquhoun M.; Tran D.; Hwang B.; Mulligan M. S.; Bryers J. D.; Girda E.; Leiserowitz G. S.; Smith Z. J.; Lam K. S. Multispectral Optical Tweezers for Biochemical Fingerprinting of CD9-Positive Exosome Subpopulations. Anal. Chem. 2017, 89 (10), 5357–5363. 10.1021/acs.analchem.7b00017. 28345878 PMC5551404

[ref27] Lee W.; Nanou A.; Rikkert L.; Coumans F. A. W.; Otto C.; Terstappen L. W. M. M.; Offerhaus H. L. Label-Free Prostate Cancer Detection by Characterization of Extracellular Vesicles Using Raman Spectroscopy. Anal. Chem. 2018, 90 (19), 11290–11296. 10.1021/acs.analchem.8b01831. 30157378 PMC6170952

[ref28] Tirinato L.; Gentile F.; Di Mascolo D.; Coluccio M. L.; Das G.; Liberale C.; Pullano S. A.; Perozziello G.; Francardi M.; Accardo A.; De Angelis F.; Candeloro P.; Di Fabrizio E. SERS Analysis on Exosomes Using Super-Hydrophobic Surfaces. Microelectron. Eng. 2012, 97, 337–340. 10.1016/j.mee.2012.03.022.

[ref29] Kerr L. T.; Gubbins L.; Weiner Gorzel K.; Sharma S.; Kell M.; McCann A.; Hennelly B. M. Raman Spectroscopy and SERS Analysis of Ovarian Tumour Derived Exosomes (TEXs): A Preliminary Study. In Biophotonics: Photonic Solutions for Better Health Care IV, SPIE Photonics Europe, Brussels, Belgium, May 8, 2014; Popp J., Tuching V. V., Matthews D. L., Pavone F. S., Garside P., Eds.; SPIE: Bellingham, WA, 2014; Vol. 9129, p 91292Q.

[ref30] Lee C.; Carney R. P.; Hazari S.; Smith Z. J.; Knudson A.; Robertson C. S.; Lam K. S.; Wachsmann-Hogiu S. 3D Plasmonic Nanobowl Platform for the Study of Exosomes in Solution. Nanoscale 2015, 7 (20), 9290–9297. 10.1039/C5NR01333J. 25939587 PMC11781986

[ref31] Stremersch S.; Marro M.; Pinchasik B. El; Baatsen P.; Hendrix A.; De Smedt S. C.; Loza-Alvarez P.; Skirtach A. G.; Raemdonck K.; Braeckmans K. Identification of Individual Exosome-Like Vesicles by Surface Enhanced Raman Spectroscopy. Small 2016, 12 (24), 3292–3301. 10.1002/smll.201600393. 27171437

[ref32] Park J.; Hwang M.; Choi B.; Jeong H.; Jung J. H.; Kim H. K.; Hong S.; Park J. H.; Choi Y. Exosome Classification by Pattern Analysis of Surface-Enhanced Raman Spectroscopy Data for Lung Cancer Diagnosis. Anal. Chem. 2017, 89 (12), 6695–6701. 10.1021/acs.analchem.7b00911. 28541032

[ref33] Shin H.; Jeong H.; Park J.; Hong S.; Choi Y. Correlation between Cancerous Exosomes and Protein Markers Based on Surface-Enhanced Raman Spectroscopy (SERS) and Principal Component Analysis (PCA). ACS Sensors 2018, 3 (12), 2637–2643. 10.1021/acssensors.8b01047. 30381940

[ref34] Ferreira N.; Marques A.; Águas H.; Bandarenka H.; Martins R.; Bodo C.; Costa-Silva B.; Fortunato E. Label-Free Nanosensing Platform for Breast Cancer Exosome Profiling. ACS Sensors 2019, 4 (8), 2073–2083. 10.1021/acssensors.9b00760. 31327232

[ref35] Yan Z.; Dutta S.; Liu Z.; Yu X.; Mesgarzadeh N.; Ji F.; Bitan G.; Xie Y. H. A Label-Free Platform for Identification of Exosomes from Different Sources. ACS Sensors 2019, 4 (2), 488–497. 10.1021/acssensors.8b01564. 30644736

[ref36] Carmicheal J.; Hayashi C.; Huang X.; Liu L.; Lu Y.; Krasnoslobodtsev A.; Lushnikov A.; Kshirsagar P. G.; Patel A.; Jain M.; Lyubchenko Y. L.; Lu Y.; Batra S. K.; Kaur S. Label-Free Characterization of Exosome *via* Surface Enhanced Raman Spectroscopy for the Early Detection of Pancreatic Cancer. Nanomedicine 2019, 16, 88–96. 10.1016/j.nano.2018.11.008. 30550805 PMC6532067

[ref37] Dong S.; Wang Y.; Liu Z.; Zhang W.; Yi K.; Zhang X.; Zhang X.; Jiang C.; Yang S.; Wang F.; Xiao X. Beehive-Inspired Macroporous SERS Probe for Cancer Detection through Capturing and Analyzing Exosomes in Plasma. ACS Appl. Mater. Interfaces 2020, 12 (4), 5136–5146. 10.1021/acsami.9b21333. 31894690

[ref38] Krafft C.; Osei E. B.; Popp J.; Nazarenko I. Raman and SERS Spectroscopy for Characterization of Extracellular Vesicles from Control and Prostate Carcinoma Patients. In Proc. SPIE 11236, Biomedical Vibrational Spectroscopy 2020: Advances in Research and Industry, San Francisco, USA, February 21, 2020; Petrich W., Huang Z., Eds.; SPIE: Bellingham, WA, 2020; Vol. 11236, p 9–13.

[ref39] Guerrini L.; Garcia-Rico E.; O’Loghlen A.; Giannini V.; Alvarez-Puebla R. A. Surface-Enhanced Raman Scattering (SERS) Spectroscopy for Sensing and Characterization of Exosomes in Cancer Diagnosis. Cancers 2021, 13 (9), 2179 10.3390/cancers13092179. 33946619 PMC8125149

[ref40] Zong S.; Wang L.; Chen C.; Lu J.; Zhu D.; Zhang Y.; Wang Z.; Cui Y. Facile Detection of Tumor-Derived Exosomes Using Magnetic Nanobeads and SERS Nanoprobes. Anal. Methods 2016, 8 (25), 5001–5008. 10.1039/C6AY00406G.

[ref41] Tian Y. F.; Ning C. F.; He F.; Yin B. C.; Ye B. C. Highly Sensitive Detection of Exosomes by SERS Using Gold Nanostar@Raman Reporter@Nanoshell Structures Modified with a Bivalent Cholesterol-Labeled DNA Anchor. Analyst 2018, 143 (20), 4915–4922. 10.1039/C8AN01041B. 30225507

[ref42] Wang Z.; Zong S.; Wang Y.; Li N.; Li L.; Lu J.; Wang Z.; Chen B.; Cui Y. Screening and Multiple Detection of Cancer Exosomes Using an SERS-Based Method. Nanoscale 2018, 10 (19), 9053–9062. 10.1039/C7NR09162A. 29718044

[ref43] Zhang W.; Jiang L.; Diefenbach R. J.; Campbell D. H.; Walsh B. J.; Packer N. H.; Wang Y. Enabling Sensitive Phenotypic Profiling of Cancer-Derived Small Extracellular Vesicles Using Surface-Enhanced Raman Spectroscopy Nanotags. ACS Sensors 2020, 5 (3), 764–771. 10.1021/acssensors.9b02377. 32134252

[ref44] Ning C. F.; Wang L.; Tian Y. F.; Yin B. C.; Ye B. C. Multiple and Sensitive SERS Detection of Cancer-Related Exosomes Based on Gold-Silver Bimetallic Nanotrepangs. Analyst 2020, 145 (7), 2795–2804. 10.1039/C9AN02180A. 32101180

[ref45] Penders J.; Pence I. J.; Horgan C. C.; Bergholt M. S.; Wood C. S.; Najer A.; Kauscher U.; Nagelkerke A.; Stevens M. M. Single Particle Automated Raman Trapping Analysis. Nat. Commun. 2018, 9 (1), 4256 10.1038/s41467-018-06397-6. 30323298 PMC6189196

[ref46] Yao M.; Wu P.; Cheng S.; Yang L.; Zhu Y.; Wang M.; Luo H.; Wang B.; Ye D.; Liu M. Investigation into the Energy Storage Behaviour of Layered α-V2O5 as a Pseudo-Capacitive Electrode Using Operando Raman Spectroscopy and a Quartz Crystal Microbalance. Phys. Chem. Chem. Phys. 2017, 19 (36), 24689–24695. 10.1039/C7CP04612J. 28861575

[ref47] Kim S. J.; Park S. J.; Kim H. Y.; Jang G. S.; Park D. J.; Park J.-Y.; Lee S.; Ahn Y. H. Characterization of Chemical Doping of Graphene by *in-Situ* Raman Spectroscopy. Appl. Phys. Lett. 2016, 108 (20), 203111 10.1063/1.4950969.

[ref48] Whittaker T. E.; Nagelkerke A.; Nele V.; Kauscher U.; Stevens M. M. Experimental Artefacts Can Lead to Misattribution of Bioactivity from Soluble Mesenchymal Stem Cell Paracrine Factors to Extracellular Vesicles. J. Extracell. Vesicles 2020, 9 (1), 1807674 10.1080/20013078.2020.1807674. 32944192 PMC7480412

[ref49] Kauscher U.; Penders J.; Nagelkerke A.; Holme M. N.; Nele V.; Massi L.; Gopal S.; Whittaker T. E.; Stevens M. M. Gold Nanocluster Extracellular Vesicle Supraparticles: Self-Assembled Nanostructures for Three-Dimensional Uptake Visualization. Langmuir 2020, 36 (14), 3912–3923. 10.1021/acs.langmuir.9b03479. 32250120 PMC7161082

[ref50] Movasaghi Z.; Rehman S.; Rehman I. U. Raman Spectroscopy of Biological Tissues. Appl. Spectrosc. Rev. 2007, 42 (5), 493–541. 10.1080/05704920701551530.

[ref51] Martin K. J.; Graner E.; Li Y.; Price L. M.; Kritzman B. M.; Fournier M. V.; Rhei E.; Pardee A. B. High-Sensitivity Array Analysis of Gene Expression for the Early Detection of Disseminated Breast Tumor Cells in Peripheral Blood. Proc. Natl. Acad. Sci. U. S. A. 2001, 98 (5), 2646–2651. 10.1073/pnas.041622398. 11226293 PMC30192

[ref52] Gonçalves A.; Charafe-Jauffret E.; Bertucci F.; Audebert S.; Toiron Y.; Esterni B.; Monville F.; Tarpin C.; Jacquemier J.; Houvenaeghel G.; Chabannon C.; Extra J. M.; Viens P.; Borg J. P.; Birnbaum D. Protein Profiling of Human Breast Tumor Cells Identifies Novel Biomarkers Associated with Molecular Subtypes. Mol. Cell. Proteomics 2008, 7 (8), 1420–1433. 10.1074/mcp.M700487-MCP200. 18426791 PMC2500227

[ref53] Eddins S. MathWorks: Rainbow Color Map Critiques: An Overview and Annotated Bibliography, 2014. https://uk.mathworks.com/content/dam/mathworks/tag-team/Objects/r/81137_92238v00_RainbowColorMap_57312.pdf (accessed 2021-06-14).

[ref54] Borland D.; Taylor R. M. Rainbow Color Map (Still) Considered Harmful. IEEE Comput. Graph. Appl. 2007, 27 (2), 14–17. 10.1109/MCG.2007.323435. 17388198

[ref55] Joint ISO/CIE Standard: Colorimetry—Part 4: CIE 1976 L*a*b* Colour Space, 2019. https://www.iso.org/obp/ui/#iso:std:iso-cie:11664:-4:ed-1:v1:en (accessed 2021-10-25).

[ref56] Liu W.; Sun Z.; Chen J.; Jing C. Raman Spectroscopy in Colorectal Cancer Diagnostics: Comparison of PCA-LDA and PLS-DA Models. J. Spectrosc. 2016, 2016, 1 10.1155/2016/1603609.

[ref57] Brozek-Pluska B.; Kopeć M.; Abramczyk H. Development of a New Diagnostic Raman Method for Monitoring Epigenetic Modifications in the Cancer Cells of Human Breast Tissue. Anal. Methods 2016, 8 (48), 8542–8553. 10.1039/C6AY02559E.

[ref58] Meksiarun P.; Aoki P. H. B.; Van Nest S. J.; Sobral-Filho R. G.; Lum J. J.; Brolo A. G.; Jirasek A. Breast Cancer Subtype Specific Biochemical Responses to Radiation. Analyst 2018, 143 (16), 3850–3858. 10.1039/C8AN00345A. 30004539

[ref59] Abramczyk H.; Imiela A. The Biochemical, Nanomechanical and Chemometric Signatures of Brain Cancer.. Spectrochim. Acta, Part A 2018, 188, 8–19. 10.1016/j.saa.2017.06.037. 28688336

[ref60] Widjaja E.; Crane N.; Chen T. C.; Morris M. D.; Ignelzi M. A.; McCreadie B. R. Band-Target Entropy Minimization (BTEM) Applied to Hyperspectral Raman Image Data. Appl. Spectrosc. 2003, 57 (11), 1353–1362. 10.1366/000370203322554509. 14658148

[ref61] Zaborowski M. P.; Balaj L.; Breakefield X. O.; Lai C. P. Extracellular Vesicles: Composition, Biological Relevance, and Methods of Study. BioScience 2015, 65 (8), 783–797. 10.1093/biosci/biv084. 26955082 PMC4776721

[ref62] Li C.; Widjaja E.; Chew W.; Garland M. Rhodium Tetracarbonyl Hydride: The Elusive Metal Carbonyl Hydride.. Angew. Chem., Int. Ed. 2002, 41 (20), 3785–3789. 10.1002/1521-3773(20021018)41:20<3785::AID-ANIE3785>3.0.CO;2-0. 12386848

[ref63] Chew W.; Widjaja E.; Garland M. Band-Target Entropy Minimization (BTEM): An Advanced Method for Recovering Unknown Pure Component Spectra. Application to the FTIR Spectra of Unstable Organometallic Mixtures. Organometallics 2002, 21 (9), 1982–1990. 10.1021/om0108752.

