# Supplementary material for: Single Particle Automated Raman Trapping Analysis of Breast Cancer Cell-Derived Extracellular Vesicles as Cancer Biomarkers
Source: ACS Nano. 2021 Nov 4;15(11):18192–205. doi: 10.1021/acsnano.1c07075 (PMC9286313; doi:10.1021/acsnano.1c07075)
Supplement: Supplementary file 1 — nn1c07075_si_001.pdf [file nn1c07075_si_001.pdf]

SUPPLEMENTARY INFORMATION

Single Particle Automated Raman Trapping Analysis of Breast Cancer Cell-Derived Extracellular Vesicles as Cancer Biomarkers

Jelle Penders[1,2,3], Anika Nagelkerke[†1,2,3], Eoghan M. Cunnane[1,2,3], Simon V. Pedersen[1,2,3]

Isaac J. Pence[1,2,3], R. Charles Coombes[4], Molly M. Stevens[1,2,3]*

[1]Department of Materials, Imperial College London, London SW7 2AZ, United Kingdom

[2]Department of Bioengineering, Imperial College London, London SW7 2AZ, United Kingdom

[3]Institute of Biomedical Engineering, Imperial College London, London SW7 2AZ, United Kingdom

[4]Department of Surgery and Cancer, Hammersmith Hospital, Imperial College, London W120HS, United Kingdom

[†]Current Address: University of Groningen, Groningen Research Institute of Pharmacy, Pharmaceutical Analysis, POB 196 XB20, NL-9700 AD Groningen, The Netherlands

*Corresponding author:

Molly Stevens,

e-mail: m.stevens@imperial.ac.uk

EV Isolation and Characterization

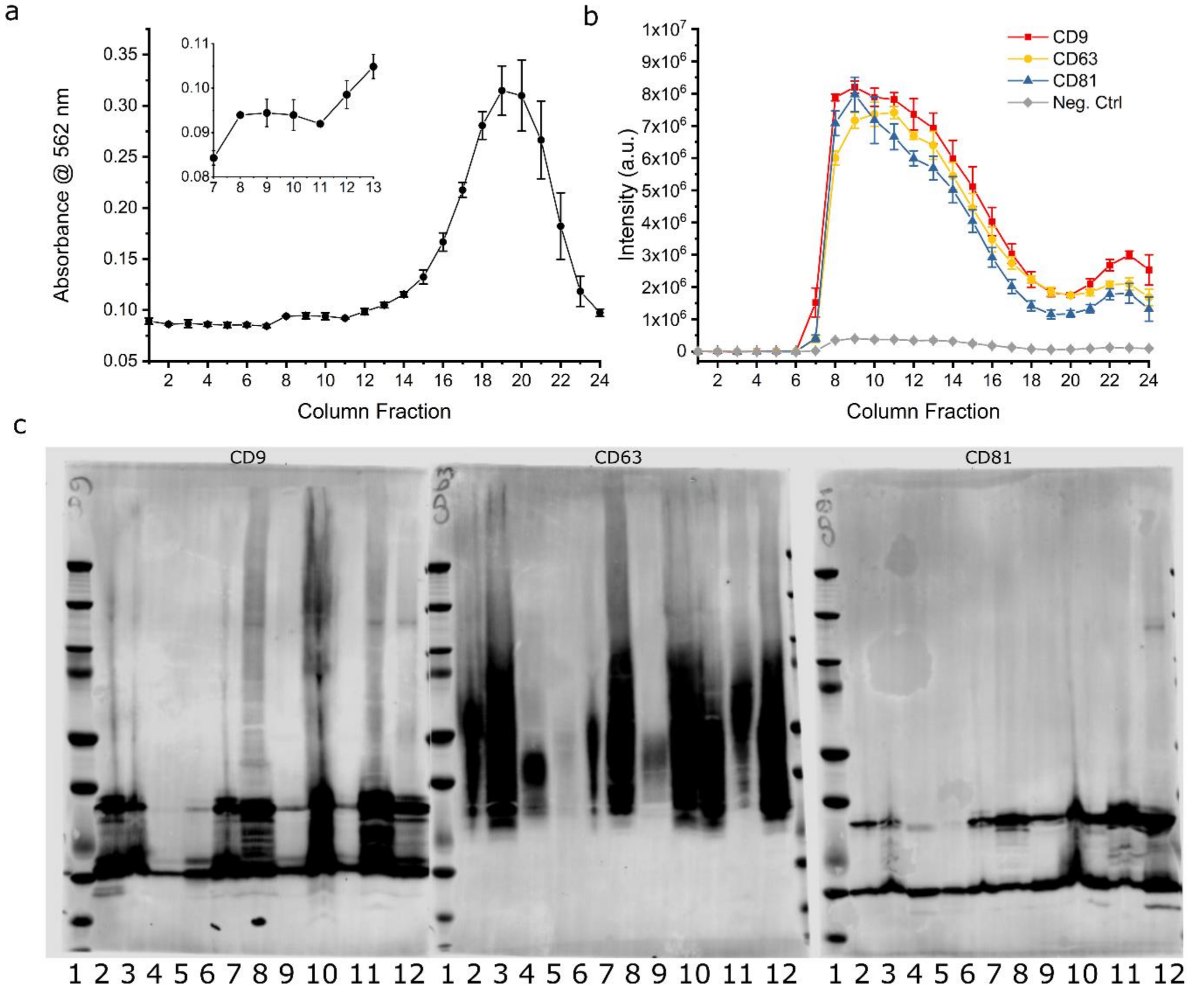

Supplementary Figure 1. BCA, Dot- and Western blot analysis. (a) BCA protein quantification (N = 3, n = 3, mean ± s.d.) of SEC column trace fractions on concentrated conditioned medium from MDA-MB-231 cells with the insert showing a closeup of the EV containing fractions. (b) Quantification of immunoblotting (dot blot) analysis for CD9, CD63 and CD81 (No primary antibody as negative control). Fractions were obtained from SEC performed on concentrated conditioned medium from MDA-MB-231 cells (N = 3, n = 1). Data presented as mean ± SD. (c) Western blot analysis for the EV markers CD9, CD63 and CD81 on purified EVs from the cell panel used in this study. Loading was as follows: (1) Marker (2) HCC1937 (3) HCC1954 (4) Hs578T (5) HuMEC (6) JIMT1 (7) MCF7 (8) MCF10A (9) MDA-MB-231 (10) MDA-MB-436 (11) MDA-MB468 (12) T47D.

The results of NTA analysis, including details regarding the concentration and size, of EVs obtained from both isolations of each cell type, are included in **Supplementary Table 1**. NTA analysis was performed on the pooled EV containing fractions (typically fractions 8-12) as obtained by size exclusion chromatography (see methods).

Supplementary Table 1: NTA data for each EV sample characterized in this study. The NTA data shows the concentration and mean particle size of the EVs (n = 3) derived from each cell type with two isolations.

Cell Type	Isolation	Concentration ± SD (particles/ml)	Mean Particle Size ± SD (nm)
HuMEC	1	9.00E+10 ± 1.39E+10	147.1 ± 52.6
	2	5.3E+10 ± 9.35E+09	114.9± 41.4
MCF10A	1	2.27E+10 ± 2.70E+09	229.4 ± 101.6
	2	4.39E+11 ± 9.05E+10	169.0 ± 69.9
MDA-MB-231	1	1.73E+11 ± 6.52E+09	154.7 ± 65.8
	2	1.47E+11 ±9.67E+09	185.4 ± 73.4
MDA-MB-436	1	1.01E+11 ± 8.37E+09	179.4 ± 73.5
	2	6.46E+10 ± 4.68E+09	153.8 ± 47.3
MDA-MB-468	1	9.18E+10 ± 4.64E+09	196.1 ± 79.4
	2	1.74E+11 ± 2.01E+10	179.4 ± 66.8
T47D	1	3.85E+10 ± 4.31E+09	160.2 ± 50.8
	2	3.21E+11 ± 7.20E+09	137.7 ± 48.6
JIMT1	1	7.27E+10 ± 5.80E+09	184.1 ± 66.6
	2	1.08E+11 ± 7.49E+09	181.7 ± 63.4
MCF7	1	1.31E+11 ± 7.73E+09	182.4 ± 67.5
	2	9.84E+10 ± 4.75E+09	181.2 ± 61.9
HCC1937	1	1.04E+11 ± 8.06E+08	184.8 ± 60.7
	2	1.25E+11 ± 5.35E+09	175.9 ± 63.3
HCC1954	1	1.30E+11 ± 4.15E+09	195.6 ± 79.1
	2	5.31E+10 ± 6.60E+09	194.4 ± 65.8
Hs578T	1	3.12E+10 ± 1.17E+09	192.6 ± 61.6
	2	2.64E+10 ± 3.72E+09	184.4 ± 72.5

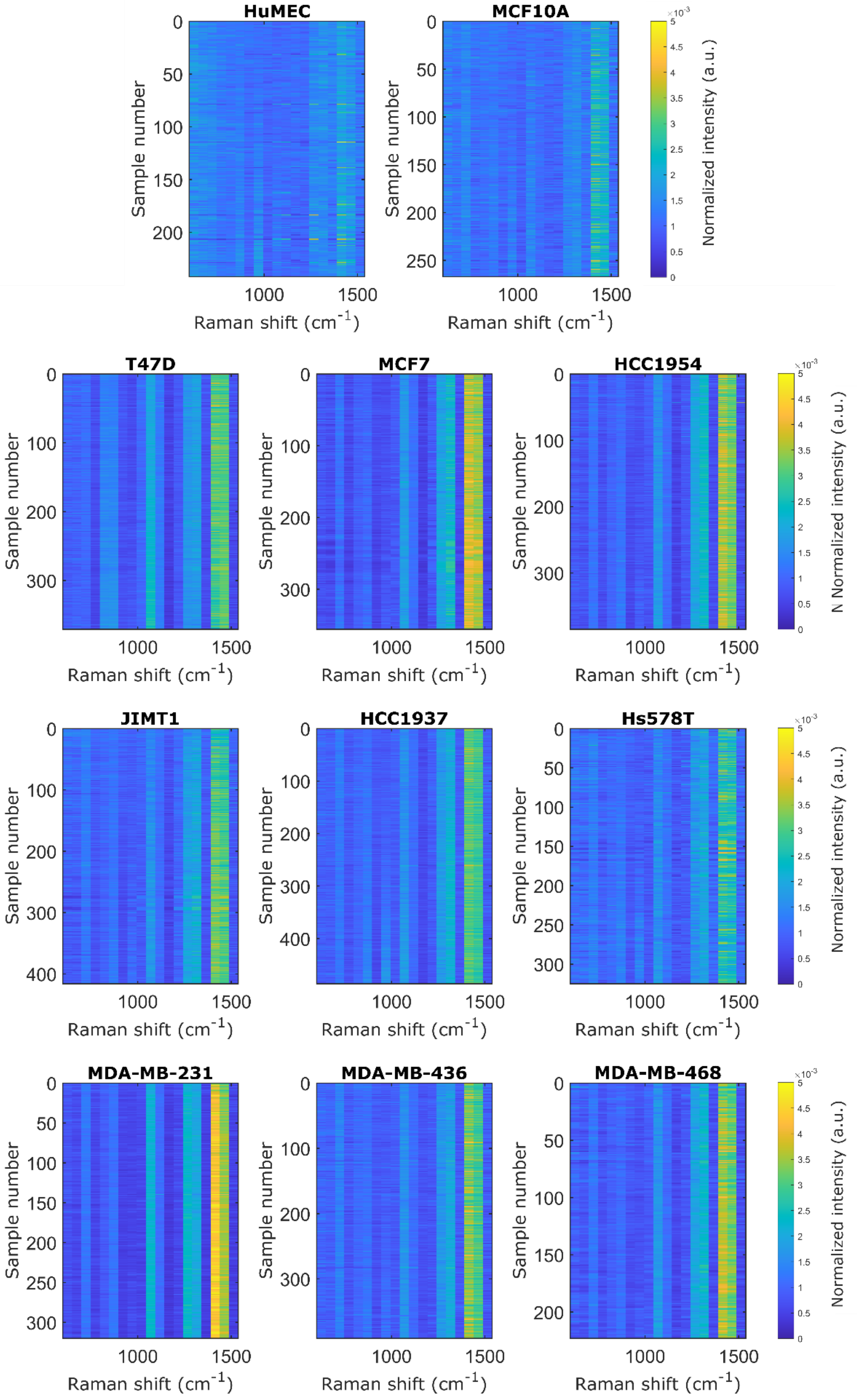

Supplementary Figure 2. DRA Analysis of Isolation 1 (20 second acquisitions). Spectra were normalized, and a fixed color scale was used for intra-sample comparison. A 50 cm^{-1} bin size was used to construct the DRA's.

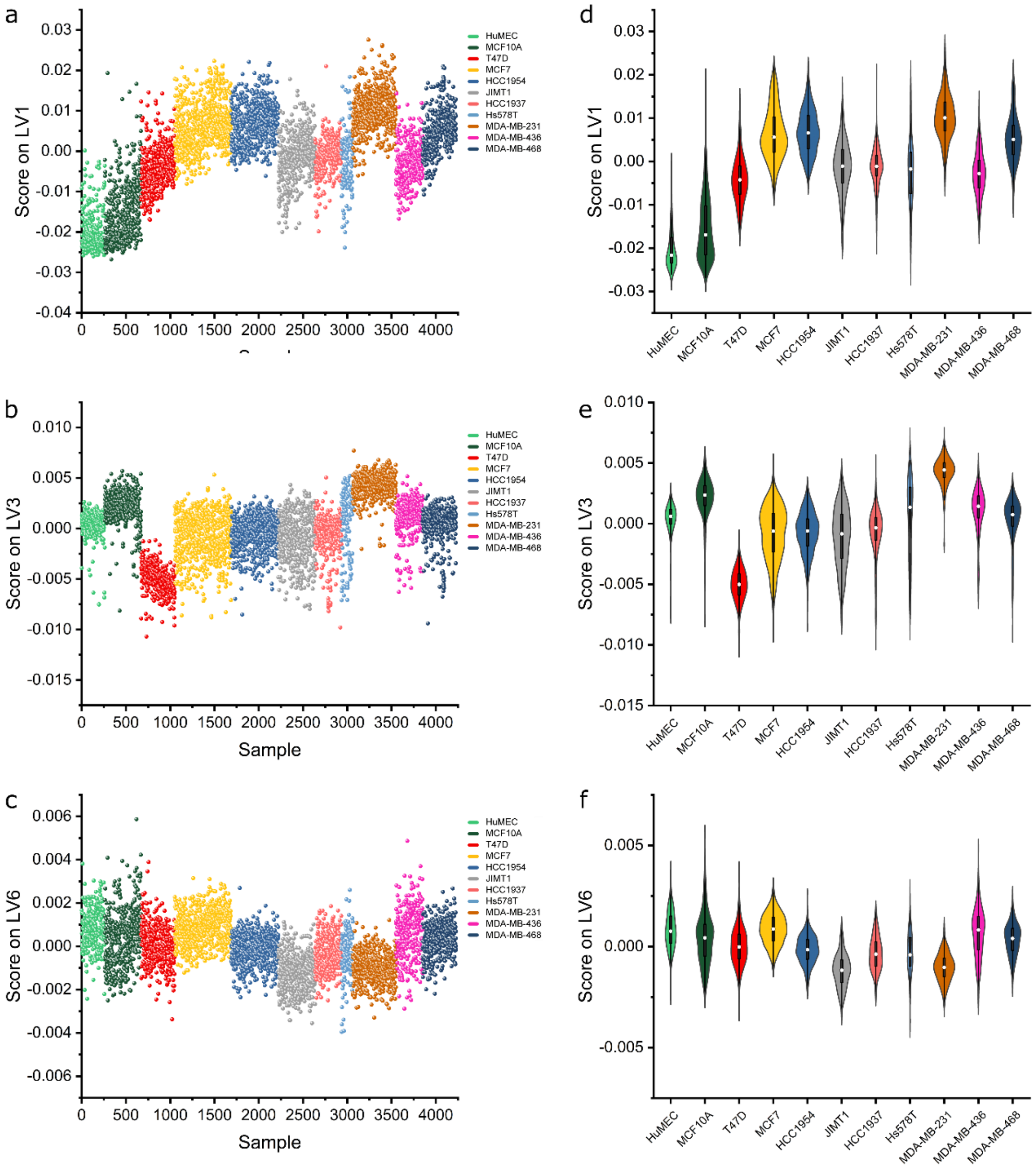

Supplementary Figure 3. PLSDA LV score plots (10 second acquisitions). Sample scores for LV 1, 3 and 6 (a-c) showing spread between classes, highlighting compositional differences based on the LV loading (Figure 4 a-c). Represented violin plots (d-f) with the median indicated by a white point and 1.5 interquartile range.

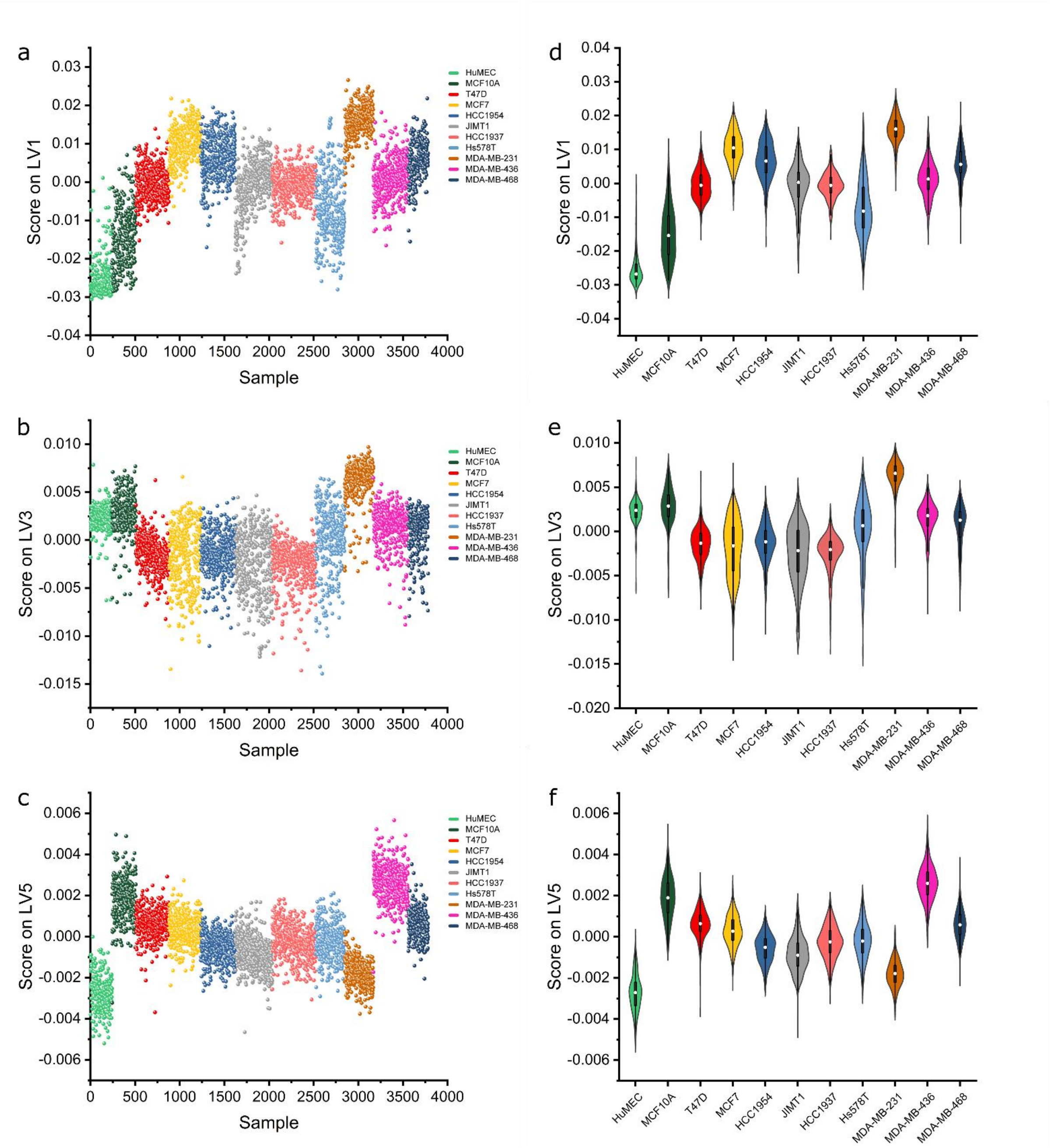

Supplementary Figure 4. PLSDA LV score plots (20 second acquisitions). Sample scores for LV 1, 3 and 5 (a-c) showing spread between classes, highlighting compositional differences based on the LV loading (Figure 4 d-f). Represented as violin (d-f) with the median indicated by a white point and 1.5 interquartile range.

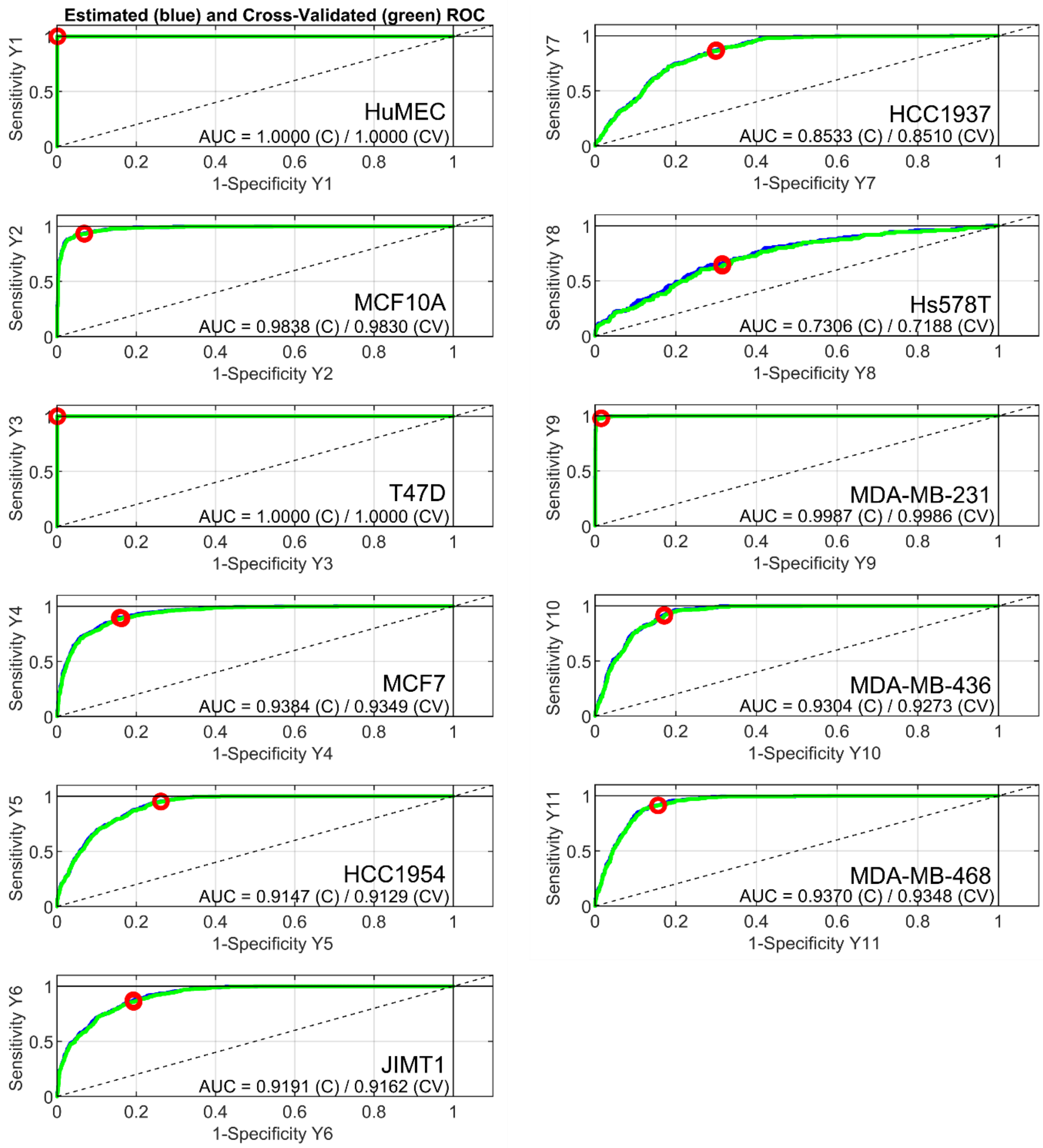

Supplementary Figure 5. Cross-validated (CV) receiver operating characteristic (ROC) curves for each class of the PLSDA model in Figure 4 a-c (10 second acquisitions). The ROC curves visualize the predictive capabilities of the model for each EV class with the area under the curve (AUC) values shown in the bottom right corner. The total average CV AUC for the model is 0.925.

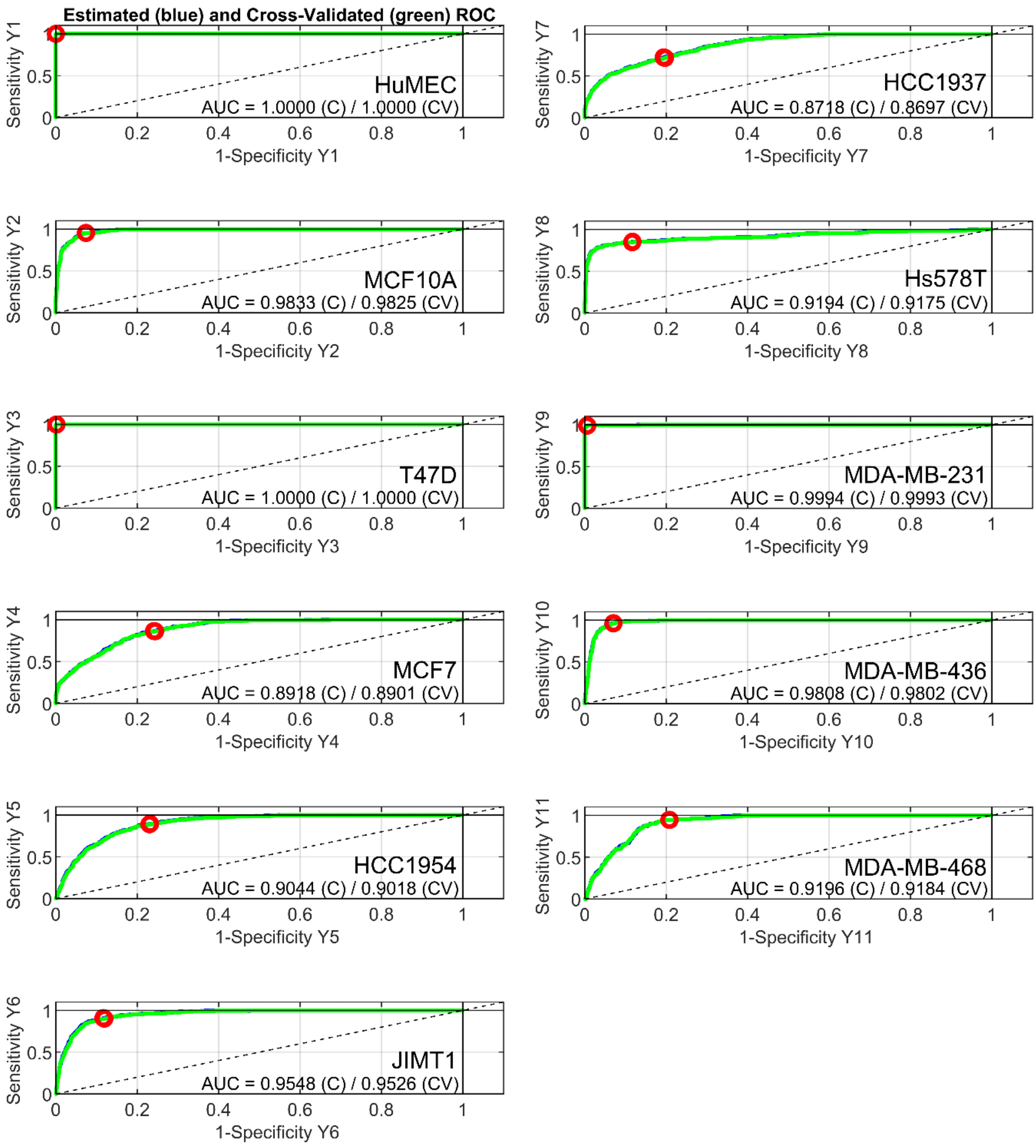

Supplementary Figure 6. Cross-validated (CV) receiver operating characteristic (ROC) curves for each class of the PLSDA model in Figure 4 d-f (20 second acquisitions). The ROC curves visualize the predictive capabilities of the model for each EV class with the area under the curve (AUC) values shown in the bottom right corner. The total average CV AUC for the model is 0.947.

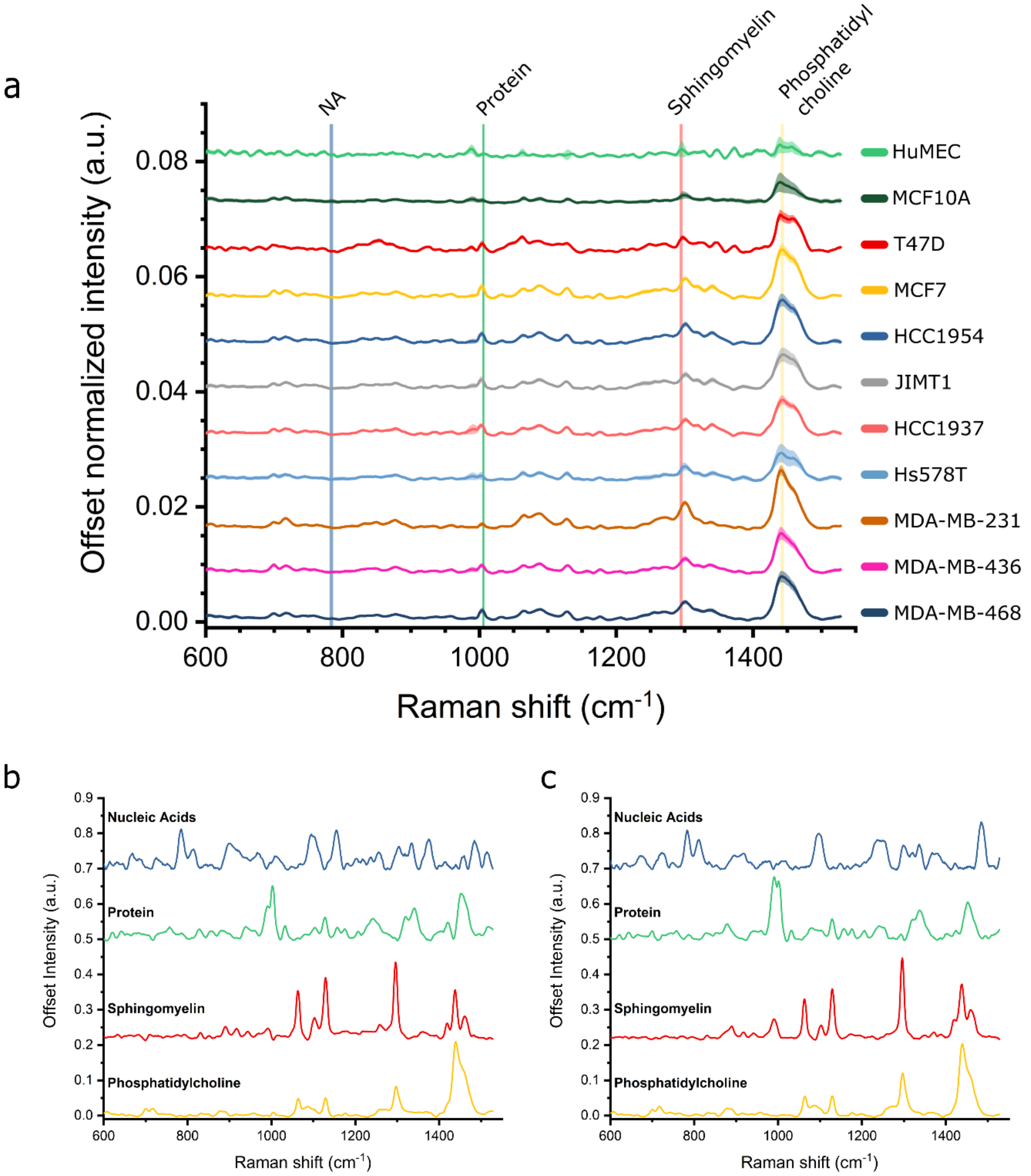

Supplementary Figure 7. Entropy minimization composition analysis of cancer and non-cancer derived EVs, selected target bands and optimized spectral components. a) Mean spectra (mean ± s.d.) of EVs derived from the 11 cell panel as measured by SPARTA® with the indicative target bands for entropy minimization analysis as shown: nucleic acids - 781.1 to 786.3 cm^{-1} (blue), protein - 1004.5 to 1007.1 cm^{-1} (green), sphingomyelin – 1292.4 to 1297.1.0 cm^{-1} (red), Phosphatidylcholine – 1440.4 to1445.0 cm^{-1} (yellow). Extracted pure component spectra for b) 10 second acquisitions (n = 3558), c) 20 second acquisitions (n = 3273).

Supplementary Table 2. Entropy minimization extracted pure component spectra band assignments. Band assignments using the compendiums tabulated by Movasaghi *et al.*[1] and Czamara *et al.*[2]

Extracted pure component spectrum compound indication	Vibration/ Compound	Raman shift (cm^{-1})
Nucleic Acids	*U, T, C ring breathing modes*	784
Nucleic Acids	*O-P-O/PO_2^-*	814
Nucleic Acids	*A, G ring breathing modes*	1336
Nucleic Acids	*A, G ring breathing modes*	1484
Protein	*C-C twisting of tyrosine*	642
Protein	*Tyrosine*	757
Protein	*Tyrosine*	856
Protein	*C-C stretching (α-helix, proteins)*	938
Protein	*Phenylalanine*	1003
Protein	*C-N stretching*	1128
Protein	*Amide III*	1243
Protein	*CH_2/CH_3 twisting and wagging in Tryptophan*	1340
Protein	*C-H deformation bands*	1452
Sphingomyelin	*C-C stretch of lipids/acyl chains*	1063
Sphingomyelin	*C-C stretch*	1103
Sphingomyelin	*C-C stretch/acyl chains*	1130
Sphingomyelin	*CH_2 twisting*	1296
Sphingomyelin	*CH_2/CH_3 scissoring and acyl chains*	1437
Phosphatidylcholine	*$N^+(CH_3)_3$ symmetric stretch/C-C-N^+ symmetric stretch in phosphatidylcholine*	717
Phosphatidylcholine	*$N^+(CH_3)_3$ antisymmetric stretch*	878
Phosphatidylcholine	*Acyl chains/C-C skeletal stretch*	1065
Phosphatidylcholine	*C-C stretch*	1088
Phosphatidylcholine	*C-C stretch of acyl backbone*	1129
Phosphatidylcholine	*=C-H deformation (phospholipids)*	1270
Phosphatidylcholine	*acyl chains/CH_2 deformation/CH_2 twisting*	1298
Phosphatidylcholine	*CH_2 scissoring*	1439

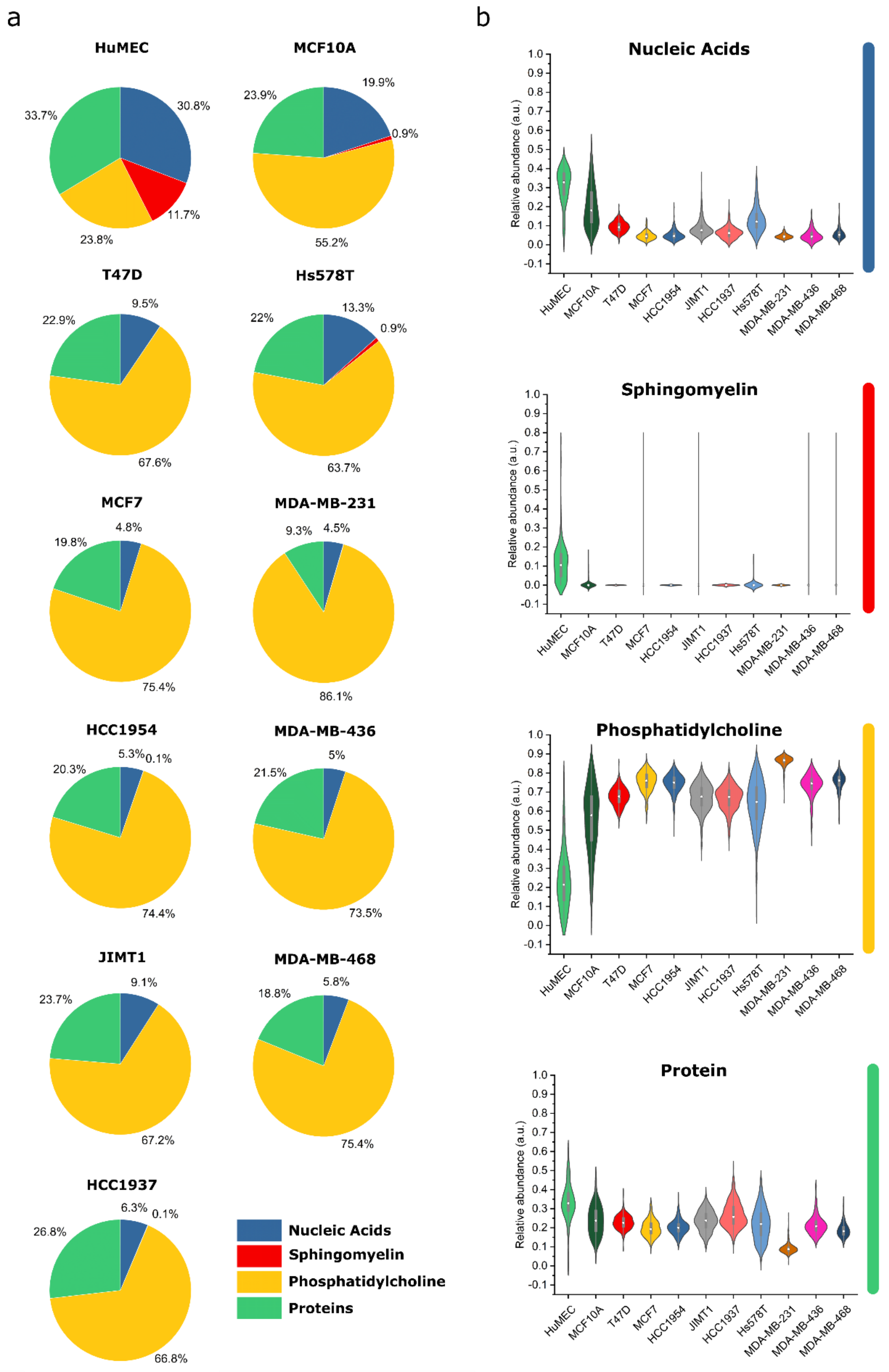

Supplementary Figure 8. Entropy minimization composition analysis of cancer and non-cancer derived EVs. (20 second acquisitions) a) Pie charts indicating the relative abundance of identified components. b) Violin plots for each component show the distribution between cancer and non-cancer EV subtypes with the median indicated by a white point and 1.5 interquartile range.

References

(1) Movasaghi, Z.; Rehman, S.; Rehman, I. U. Raman Spectroscopy of Biological Tissues. *Appl. Spectrosc. Rev.* **2007**, *42* (5), 493–541.

(2) Czamara, K.; Majzner, K.; Pacia, M. Z.; Kochan, K.; Kaczor, A.; Baranska, M. Raman Spectroscopy of Lipids: A Review. *J. Raman Spectrosc.* **2015**, *46* (1), 4–20.
